# Supplementary material for: METTL9 sustains vertebrate neural development primarily via non-catalytic functions
Source: Nat Commun. 2025 Aug 1;16:7051. doi: 10.1038/s41467-025-62414-5 (PMC12313917; doi:10.1038/s41467-025-62414-5)

Supplementary Information

METTL9 sustains vertebrate neural development primarily via non-catalytic functions

Authors list

Azzurra Codino[1], Luca Spagnoletti[1], Claudia Olobardi[2], Alessandro Cuomo[3], Helena Santos-Rosa[4], Martina Palomba[5], Natasha Margaroli[5], Stefania Girotto[5], Rita Scarpelli[5], Shi-Lu Luan[6,#], Eleonora Crocco[7], Paolo Bianchini[8], Andrew J. Bannister[4], Stefano Gustincich[1], Tony Kouzarides[4], Riccardo Rizzo[9], Isaia Barbieri[6,10], Federico Cremisi[7], Robert Vignali[2] and Luca Pandolfini[1,✉]

Affiliations

[1] Italian Institute of Technology (IIT), Center for Human Technologies, Via Enrico Melen 83, 16152 Genoa, Italy.

[2] Department of Biology, University of Pisa, via Luca Ghini 13, 56126 Pisa, Italy.

[3] Department of Experimental Oncology, IEO, European Institute of Oncology IRCCS, Milan, 20139, Italy.

[4] The Wellcome Trust/Cancer Research UK Gurdon Institute and Department of Pathology, University of Cambridge, Tennis Court Road, Cambridge, CB2 1QN, United Kingdom.

[5] Italian Institute of Technology (IIT), Center for Convergent Technologies, Via Morego 30, 16163 Genoa, Italy.

[6] University of Cambridge, Dep. of Pathology, 10 Tennis Court Road, Cambridge CB2 1QP, United Kingdom.

[7] BIO@SNS, Scuola Normale Superiore di Pisa, Via Giuseppe Moruzzi, 56124 Pisa, Italy.

[8] NIC@IIT, Italian Institute of Technology (IIT), Via Enrico Melen 83, 16152 Genoa, Italy.

[9] Institute of Nanotechnology, National Research Council (CNR-NANOTEC), Campus Ecotekne, Via Monteroni, 73100, Lecce, Italy.

[10] Department of molecular biotechnology and health sciences, Molecular Biotechnology Center, University of Turin, Via Nizza 52, 10126, Turin, Italy.

[#]Present address: Protein & Nucleic Acid Chemistry Division, MRC Laboratory of Molecular Biology, Cambridge, United Kingdom.

✉: luca.pandolfini@iit.it

Table of contents

Supplementary Figures 1-12 and legends

Uncropped gels, blots and micrographs

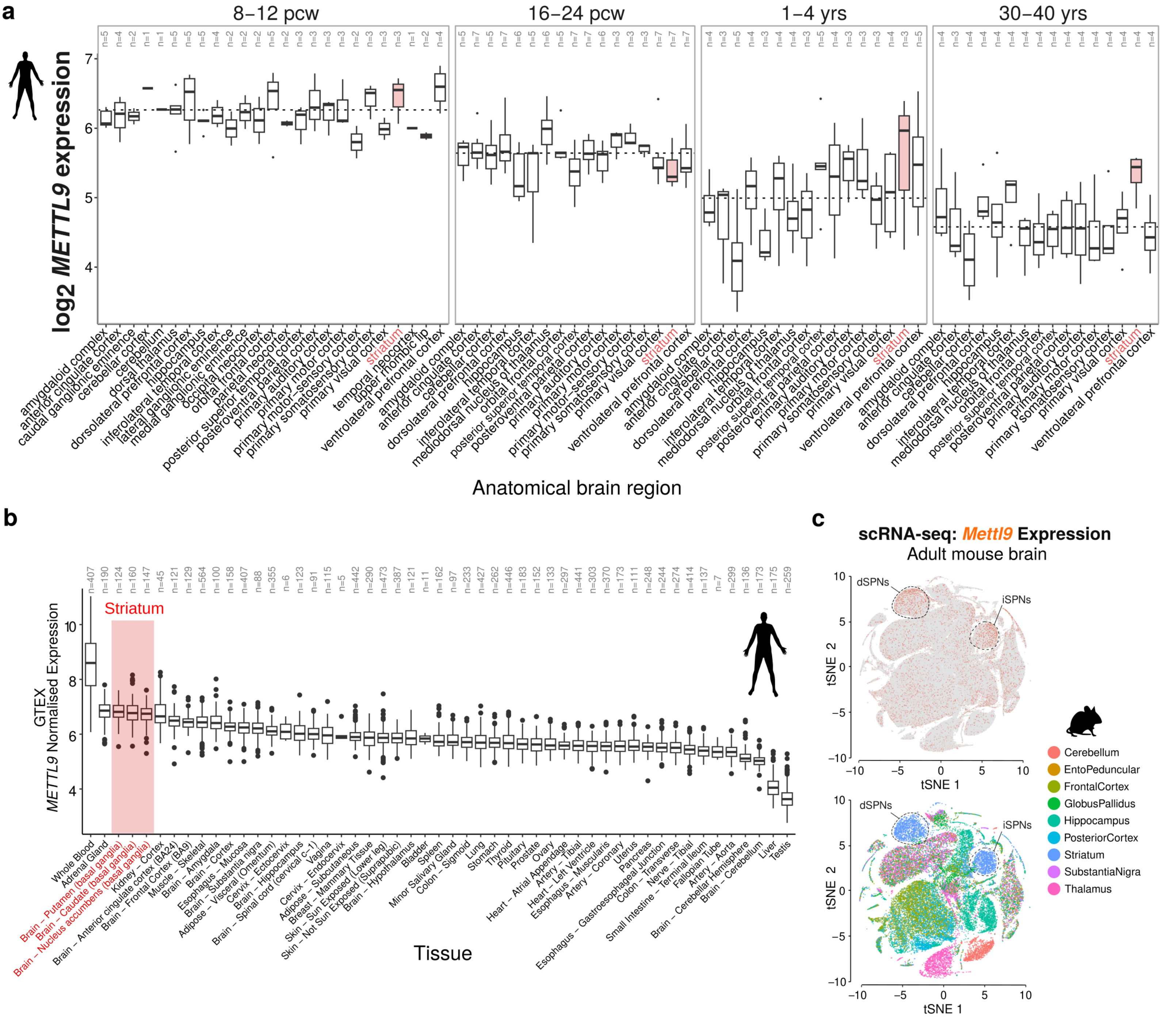
a
8–12 pcw
16–24 pcw
1–4 yrs
30–40 yrs
log2 METTL9 expression
Anatomical brain region
striatum
b
Striatum
GTEX METTL9 Normalised Expression
Tissue
Whole Blood
Adrenal Gland
Brain – Putamen (basal ganglia)
Brain – Caudate (basal ganglia)
Brain – Nucleus accumbens (basal ganglia)
Testis
c
scRNA-seq: Mettl9 Expression
Adult mouse brain
dSPNs
iSPNs
tSNE 1
tSNE 2
Cerebellum
EntoPeduncular
FrontalCortex
GlobusPallidus
Hippocampus
PosteriorCortex
Striatum
SubstantiaNigra
Thalamus

Supplementary Fig. 1

Mettl9 expression in human brain development and mouse adult brain.

a Boxplot showing re-analysis of *METTL9* expression (log_2) in pre-natal (8-12 and 16-24 post conception weeks; pcw), and post-natal (1-4 and 30-40 years; yrs) brain regions (BrainSpan Atlas of the Developing Human Brain). The striatum is shown in pink. Sample size (n) is shown for each brain region, on the top of the graph.
b Re-analysis of normalised *METTL9* expression within RNA-seq datasets from human tissues (GTEx); the striatum (putamen, caudate, nucleus accumbens) is highlighted in pink and red. Sample size (n) is shown for each tissue, on the top of the graph.
c Re-analysis of scRNA-seq data (Saunders et al. 2018) of adult mouse brain; on the left, t-distributed stochastic neighbor embedding (tSNE) plot with *Mettl9* expression (orange) in direct and indirect spiny projection neurons (dSPNs and iSPNs).

Supplementary Figure 2 ***Codino et al.***

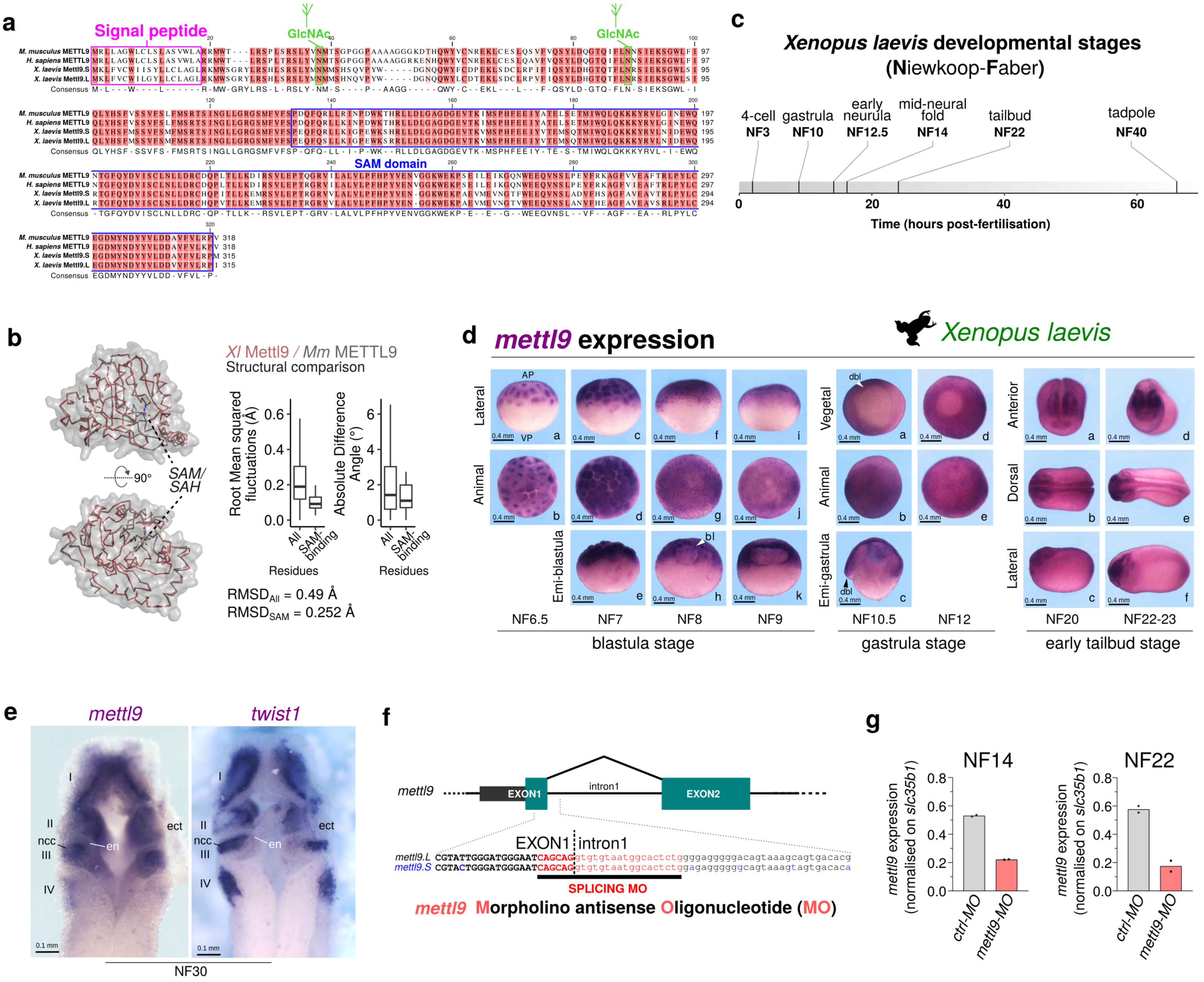

Supplementary Fig. 2
mettl9* expression and knock down strategy in *X. laevis.

a Protein alignment of mouse, human, and *X. laevis* S and L (Short and Long chromosome) METTL9/Mettl9. The signal peptide predicted site, the predicted N-Acetylglucosamine (GlcNAc) deposition sites, and the SAM domain are highlighted.
b Structural comparison of *X. laevis* (*Xl*) Mettl9 and *M. musculus (Mm)* METTL9 proteins (the backbones of the AlphaFold predictions are superimposed in red and gray, respectively). Boxplots on the right show the distributions of root mean squared fluctuations and pseudo-torsion angle deviations for all residues (All; n=250) and amino acids engaged in SAM binding (SAM-binding, including D151/G153; n=19). Global root mean squared deviations (RMSD) are indicated in the lower part.
c Main developmental stages of *X. laevis.*
d *mettl9* mRNA expression at blastula (NF 6.5-9), gastrula (10.5-12) and early tailbud (NF20-23) stages, shown by RNA WISH. AP: Animal Pole; VP: Vegetal Pole; bl: blastocoel; dbl: dorsal blastopore lip. Scale bar is 0.4 mm. Images are representative of N=30 embryos analysed per probe/stage.
e *mettl9* and *twist1* expression at stage NF30 in horizontally sectioned embryos, shown by WISH. The core of the pharyngeal arches (I-IV) (neural crest cells, ncc), endoderm (en) and ectoderm (ect) are shown. Scale bar is 0.1 mm. Images are representative of N=15 embryos analysed per probe.
f *mettl9* Morpholino antisense Oligonucleotide (MO) targeting the exon1-intron1 junction of *X. laevis mettl9* pre-mRNA.
g Relative expression of *mettl9* mRNA (by qPCR) normalised on *slc35b1*, at NF14 and NF22 stages, in control (*ctrl*-) and *mettl9*-MO embryos. N=2, where each sample is a pool of 5 embryos.

a

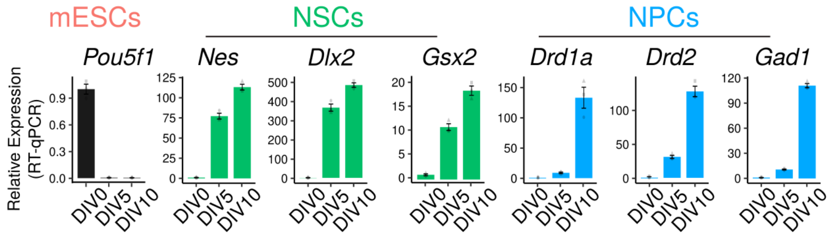

b

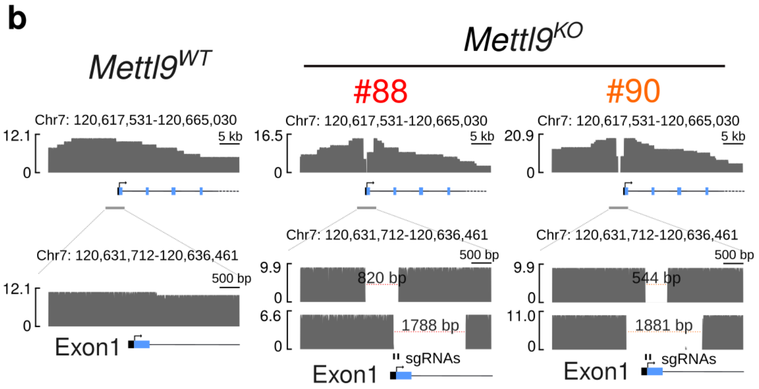

c

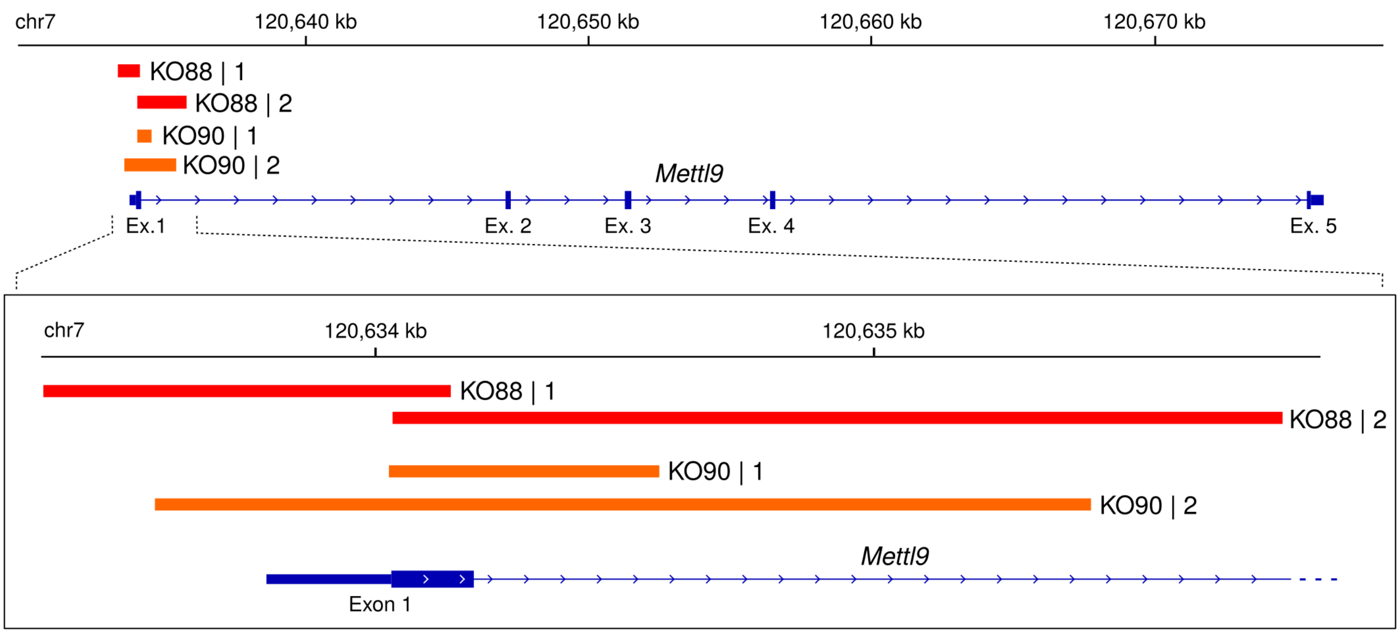

Deletion	Annotation	Impact	HGVS.c	HGVS.p	Description
**KO88 | 1**	frameshift_variant, start_lost	HIGH	c.-700_119del	p.Met1fs	A deletion from the promoter (700 bases upstream the 5'UTR) through coding position 119, causing the deletion from Met1 to Pro40 and a frameshift afterwards.
**KO88 | 2**	start_lost, splice_donor_variant, conservative_inframe_deletion, splice_region_variant, intron_variant	HIGH	c.1_165+1622del	p.Met1_Gln55del	A deletion from the start codon (included) through 1622 bases of the first intron, causing the loss of all the initial AUGs and Met1-Gln55
**KO90 | 1**	start_lost, conservative_inframe_deletion, splice_region_variant	HIGH	c.-6_165+372del	p.Met1_Gln55del	A deletion from 6 bases upstream the start codon through 372 bases of the first intron, causing the loss of all the initial AUGs and Met1-Gln55
**KO90 | 2**	exon_loss_variant, splice_region_variant	HIGH	c.-476_165+1239del		A deletion from the promoter (476 bases upstream the 5'UTR) through 1239 bases of the first intron, causing the loss of the whole first exon

d

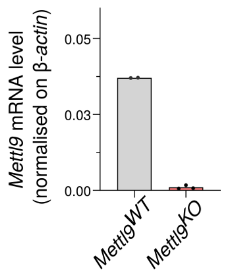

e

Putative Off-Target site number (allowing up to 5 mismatches)				
**Total**		**Overlap. with any variant, including those present also in parental E14**	**Overlap. with a *Mettl9*^{KO} cell line-specific variant**	**MAX Proximity to variant (bp)**
1075	**SNV**	0	0	100
		4	0	1000
		20	0	10000
	**SV**	0	0	100
		0	0	1000
		0	0	10000

Supplementary Fig. 3
Validation of the *Mettl9*^{KO} mESC lines.

a Relative mRNA expression level (by qPCR) of mESC, NSCs or NPCs marker genes at DIV0, DIV5 and DIV10 during mESC neural differentiation. N=3 differentiation experiments; error bars represent mean ± SD.
b Validation of the *Mettl9* (Exon1) deletions by long-read sequencing, in the *Mettl9*^{KO} mESC lines (#88 and #90), generated by CRISPR/Cas9. Diagrams show the read coverage (dark gray histogram) across the deletion regions. Chr: chromosome.
c Genomic diagram showing the overall *Mettl9* gene structure (top) and a close-up (bottom) of the exact positions of the biallelic deletions (del) in *Mettl9*^{KO} #88 and #90 cell lines (red and orange, respectively). The lower table provides a detailed annotation of the functional consequences due to each deletion, including their nomenclature, which follows the HGVS (Human Genome Variation Society) standard.
d Relative *Mettl9* mRNA expression level (qPCR) in *Mettl9*^{WT} (N=2) and *Mettl9*^{KO} (N=3) NPCs.
e Table summarising the total number of putative off-target (OT) sites (OT; left column), and the number of OT overlapping either ubiquitous (center column) or *Mettl9*^{KO}-specific variants (SNV: single-nucleotide variants; SV: structural variants). These figures are calculated taking into consideration a 100, 1000 and 10000-bp window around each putative OT.

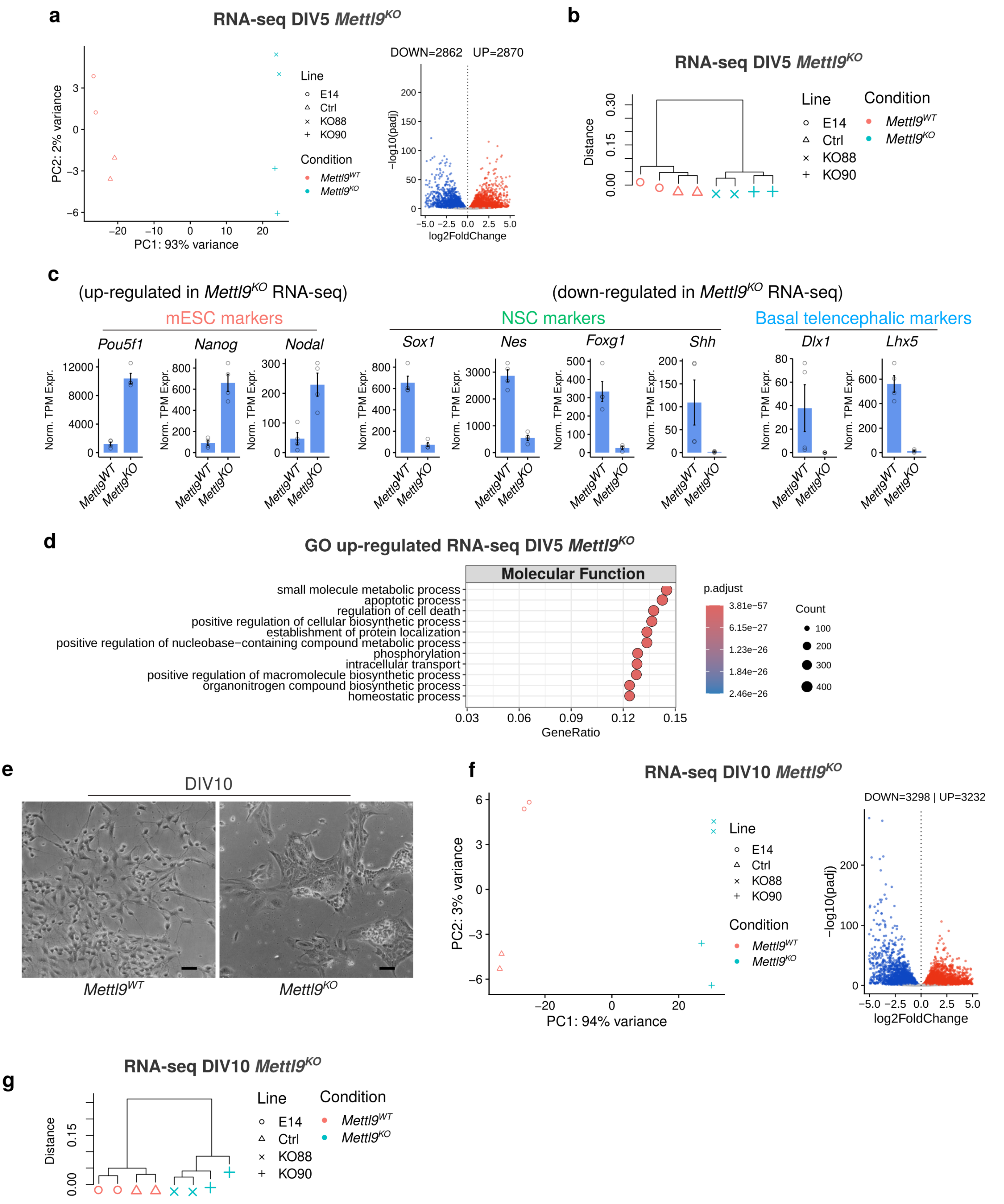
a
RNA-seq DIV5 Mettl9KO
PC2: 2% variance
PC1: 93% variance
Line
E14
Ctrl
KO88
KO90
Condition
Mettl9WT
Mettl9KO
DOWN=2862 UP=2870
−log10(padj)
log2FoldChange
b
RNA-seq DIV5 Mettl9KO
Distance
c
(up-regulated in Mettl9KO RNA-seq)
(down-regulated in Mettl9KO RNA-seq)
mESC markers
NSC markers
Basal telencephalic markers
Pou5f1
Nanog
Nodal
Sox1
Nes
Foxg1
Shh
Dlx1
Lhx5
Norm. TPM Expr.
Mettl9WT
Mettl9KO
d
GO up-regulated RNA-seq DIV5 Mettl9KO
Molecular Function
small molecule metabolic process
apoptotic process
regulation of cell death
positive regulation of cellular biosynthetic process
establishment of protein localization
positive regulation of nucleobase−containing compound metabolic process
phosphorylation
intracellular transport
positive regulation of macromolecule biosynthetic process
organonitrogen compound biosynthetic process
homeostatic process
GeneRatio
p.adjust
3.81e−57
6.15e−27
1.23e−26
1.84e−26
2.46e−26
Count
100
200
300
400
e
DIV10
Mettl9WT
Mettl9KO
f
RNA-seq DIV10 Mettl9KO
PC2: 3% variance
PC1: 94% variance
DOWN=3298 | UP=3232
−log10(padj)
log2FoldChange
g
RNA-seq DIV10 Mettl9KO
Distance
Line
E14
Ctrl
KO88
KO90
Condition
Mettl9WT
Mettl9KO

Supplementary Fig. 4
Phenotypic characterisation of the *Mettl9^{KO}* mESC lines at DIV5 and DIV10.

a Principal component analysis (PCA) plot (left) and volcano plot (right) of Differentially Expressed Genes (DEG) in *Mettl9^{WT}* and *Mettl9^{KO}* NSCs (DIV5 RNA-seq). The Y axis of volcano plot represents log10 adjusted (BH-corrected) p-values; two-tailed negative binomial Wald test.
b Hierarchical clustering analysis showing very high consistency in global gene expression values between *Mettl9^{WT}* cells (i.e. the parental E14 and a non-edited clonal control line), and between *Mettl9^{KO}* clones (i.e. #88 and #90) at DIV5, as indicated by the low similarity distance values within each of these groups.
c Normalised Transcript per million (TPM) expression of mESC (light red), NSCs (green) and NPCs (light blue) marker genes. Error bars represent mean ± SE of N=4.
d Top (10) GO Molecular Function terms up-regulated in *Mettl9^{KO}* RNA-seq (DIV10). Colour scale shows adjusted p-values (Benjamini-Hochberg correction) of the Hypergeometric test.
e Representative brightfield images of *Mettl9^{WT}* (N=3) and *Mettl9^{KO}* (N=5) NPCs at DIV10. Scale bar is 20 μm. All fields of view were taken from 2 independent differentiation experiments.
f PCA plot (left) and volcano plot (right) of Differentially Expressed Genes (DEG) in *Mettl9^{WT}* and *Mettl9^{KO}* NPCs (RNA-seq DIV10). The Y axis of volcano plot represents log10 (BH-corrected) p-values; two-tailed negative binomial Wald test.
g Hierarchical clustering analysis for cells at DIV10, following the same criteria as in panel **b** (DIV5), confirming a high consistency within *Mettl9^{WT}* and *Mettl9^{KO}* cell groups also at a later time point of differentiation.

Supplementary Figure 5

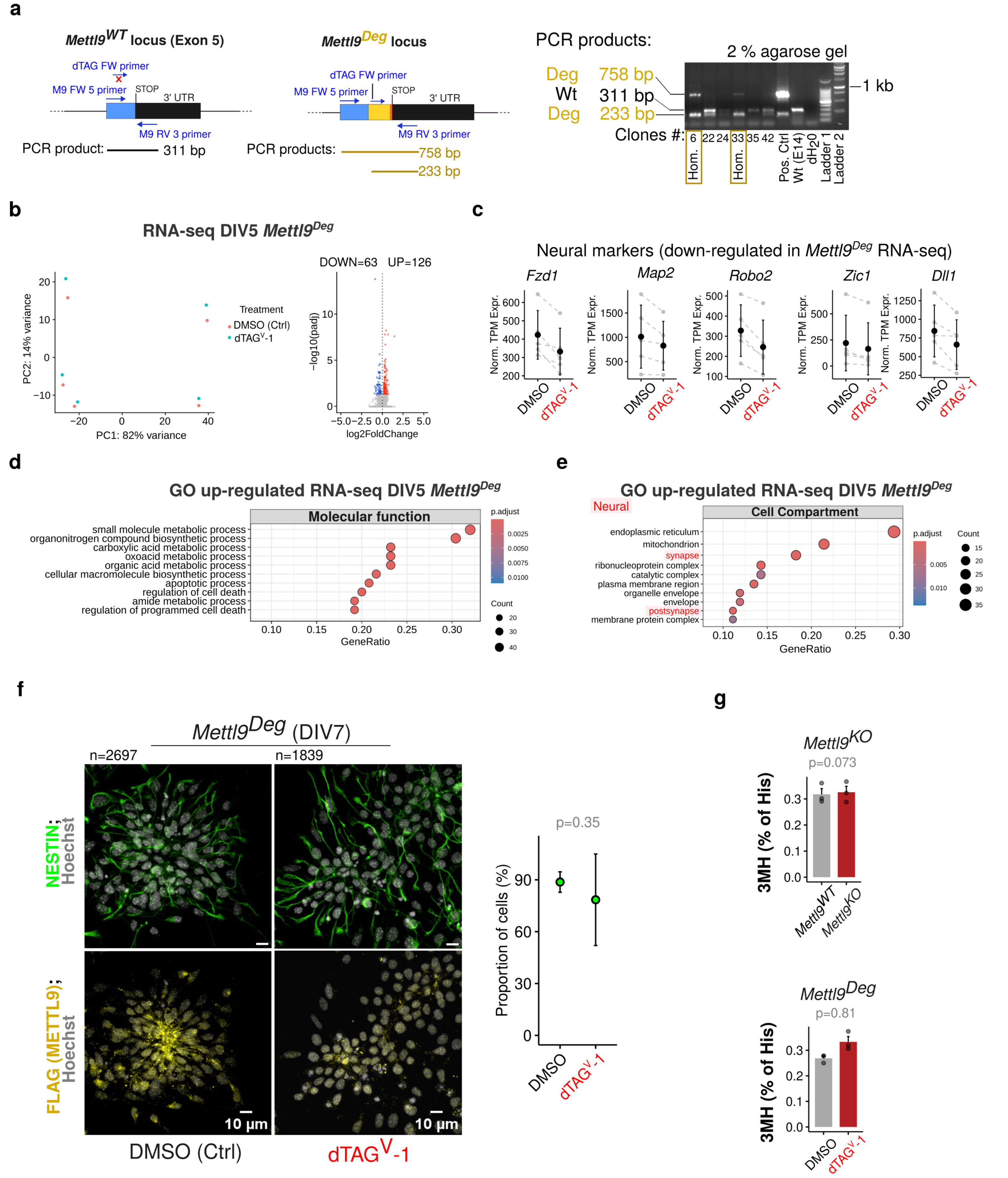

Supplementary Fig. 5

Characterization and transcriptomic analysis of the *Mettl9^{Deg}* mESC line.

a Genotyping strategy used to screen *Mettl9^{Deg}* mESC clones. On the left, the schematic depicts the amplification of the 311 bp PCR product from the *Mettl9^{WT}* locus (where only 2 primers out of three anneal); the central part of the panel shows that PCR amplification of the *Mettl9^{Deg}* locus generates 2 products of 758 bp and 233 bp. On the right, an agarose gel shows examples of PCR products from homozygous (Hom.) *Mettl9^{Deg}* ESC clones (#6 and #33, rectangles) with 2 bands (the highest at 758 bp and lowest at 233 bp). The positive control (Pos. Ctrl) is the targeting vector; PCR-amplification of untargeted *Mettl9^{WT}* locus generates only one band (311 bp), as shown in the WT (E14) lane. DNA ladder 1 and 2: 100 bp and 1 kb ladders, respectively. Independent *Mettl9^{Deg}* PCRs were performed multiple times, with similar PCR strategies.
b PCA (left) and volcano plot (right) of Differentially Expressed Genes (DEG) in *Mettl9^{Deg}* NPCs (DIV5 RNA-seq). The Y axis of volcano plot represents log10 adjusted (BH-corrected) p-values; two-tailed negative binomial Wald test.
c Normalised Transcripts per million (TPM) expression of neural marker genes from *Mettl9^{Deg}* RNA-seq (DIV5). Error bars represent mean ± SD of N=5.
d Top 10 up-regulated GO Molecular function terms in *Mettl9^{Deg}* (RNA-seq, DIV5).
e Top up-regulated Cellular Component GO terms in *Mettl9^{Deg}* RNA-seq experiment (RNA-seq, DIV5). Colour scale in (**d**),(**e**) shows adjusted p-values (Benjamini-Hochberg correction) of the Hypergeometric test.
f Representative immunofluorescence (IF) images of DMSO- and dTAG^{V}-1 treated *Mettl9^{Deg}* NSCs (DIV7), stained with an anti-NESTIN antibody (green), Hoechst (grey), and anti-FLAG antibody (yellow). Scale bar is 10 μm. Relative quantification of NESTIN signal on the right (t-test, two-sided); error bars are mean ± SD. Number of cells (n) counted is shown above each panel; 7 representative fields of view were analysed per condition.
g Relative bulk 3MH levels (% of total histidine) quantified by mass spectrometry in *Mettl9^{KO}* and dTAG^{V}-1 treated *Mettl9^{Deg}* compared to *Mettl9^{WT}* and DMSO-treated *Mettl9^{Deg}* (respectively) NSCs at DIV6. P-values of the t-test (two-sided) are shown in grey ($P \geq 0.05$); error bars show mean + SE, N=3.

Supplementary Figure 6

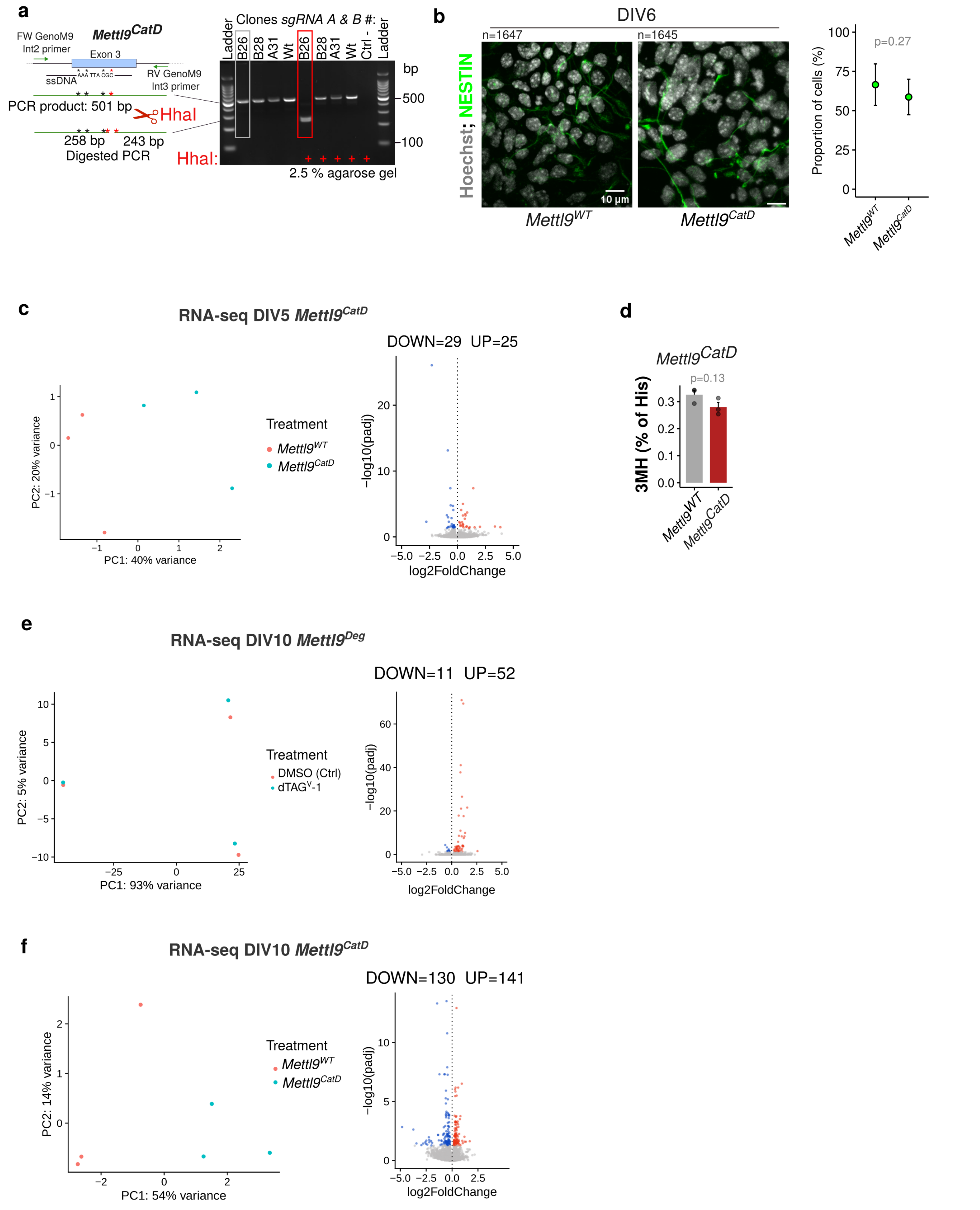

Supplementary Fig. 6

Characterisation of the *Mettl9*^{CatD} mESC line and of METTL9 catalytic dependent roles in neural development.

a Genotyping strategy adopted to screen *Mettl9*^{CatD} mESC clones. On the left, the schematic depicts the PCR amplification of the targeted *Mettl9*^{CatD} locus (asterisks are the 4 desired mutations), which generate a 501 bp product. Upon HhaI addition, only mutated PCR products (harboring the fourth mutation, red asterisk) are cut and generate 2 DNA fragments of 258 bp and 243 bp. On the right, an agarose gel shows the 501 bp PCR product from a homozygous *Mettl9*^{CatD} clone (#B26, rectangle), which is shifted after HhaI digestion (double band, 258 and 243 bp). HhaI-resistant PCR products identify DNA from *Mettl9*^{WT} clones (e.g. #B28). bp: base pair. This result was confirmed also by an independent PCR strategy.
b IF images of *Mettl9*^{CatD} DIV5 NSCs stained with anti-NESTIN antibody and Hoechst. Scale bar is 10 µm. Relative quantification of NESTIN signal on the right. The numbers of cells counted are shown above each panel. T-test (two-sided); error bars are mean ± SD. Representative fields of view analysed: N=7 (*Mettl9*^{WT}) and N=6 (*Mettl9*^{CatD}), respectively.
c Principal Component Analysis (PCA) (left) and volcano plot (right) of differentially expressed genes in *Mettl9*^{WT} and *Mettl9*^{CatD} NSCs (DIV5 RNA-seq).
d Relative 3MH levels (% of histidine) in *Mettl9*^{WT} (grey) and *Mettl9*^{CatD} (red) NSCs quantified by mass spectrometry. P-value of the t-test is shown in grey ($P \geq 0.05$); error bars show mean + SE, N=3.
e PCA (left) and volcano plot (right) of differentially expressed genes in Ctrl (DMSO-) and dTAG^{V}-1-treated *Mettl9*^{Deg} NPCs (DIV10 RNA-seq).
f PCA (left) and volcano plot (right) of differentially expressed genes in *Mettl9*^{WT} and *Mettl9*^{CatD} NPCs (DIV10 RNA-seq).
For (**c**), (**e**), (**f**): The Y axis of volcano plot represents log10 adjusted (BH-corrected) p-values; two-tailed negative binomial Wald test.

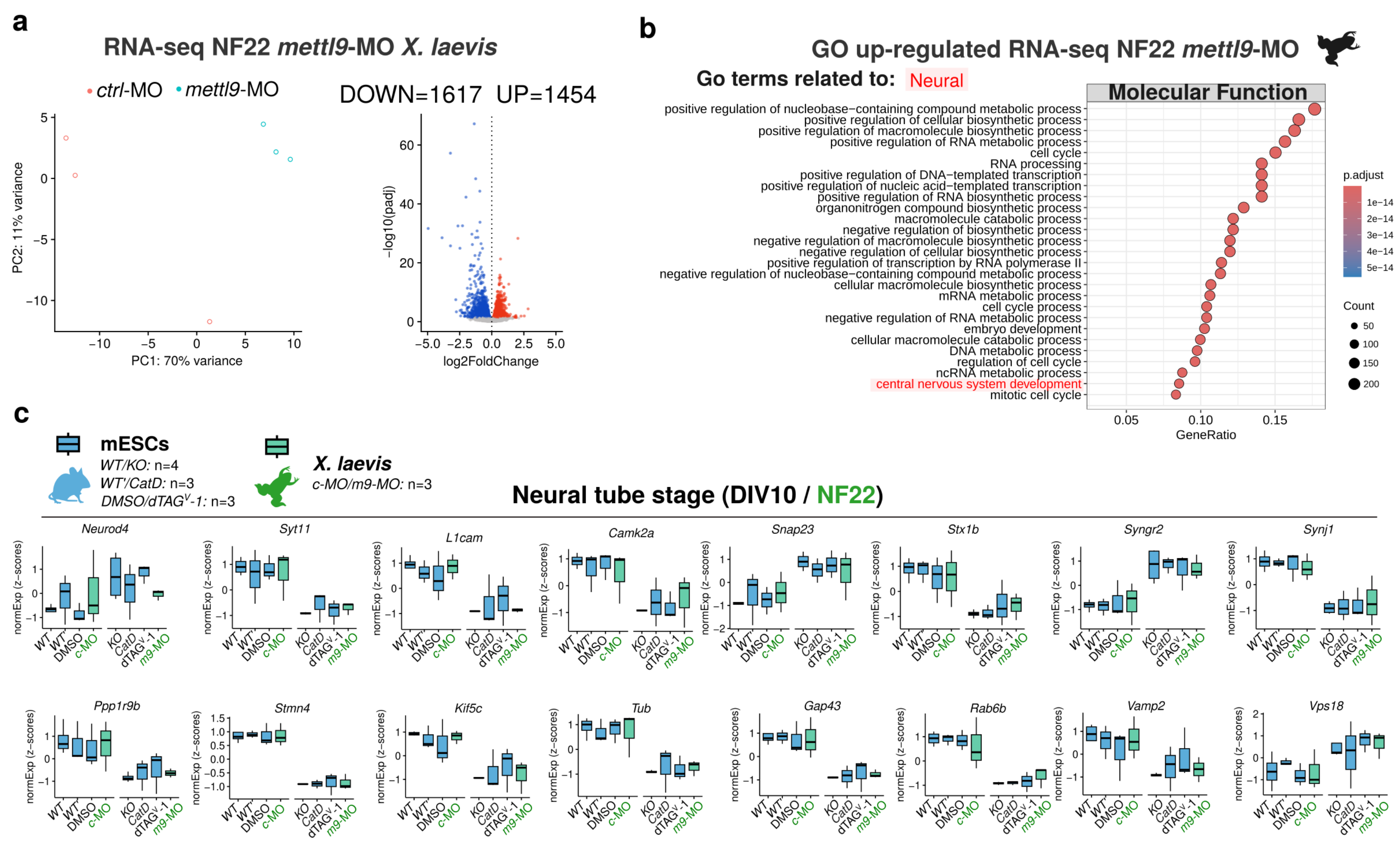
a
RNA-seq NF22 mettl9-MO X. laevis
ctrl-MO
mettl9-MO
PC1: 70% variance
PC2: 11% variance
DOWN=1617 UP=1454
log2FoldChange
−log10(padj)
b
GO up-regulated RNA-seq NF22 mettl9-MO
Go terms related to: Neural
Molecular Function
positive regulation of nucleobase−containing compound metabolic process
positive regulation of cellular biosynthetic process
positive regulation of macromolecule biosynthetic process
positive regulation of RNA metabolic process
cell cycle
RNA processing
positive regulation of DNA−templated transcription
positive regulation of nucleic acid−templated transcription
positive regulation of RNA biosynthetic process
organonitrogen compound biosynthetic process
macromolecule catabolic process
negative regulation of biosynthetic process
negative regulation of macromolecule biosynthetic process
negative regulation of cellular biosynthetic process
positive regulation of transcription by RNA polymerase II
negative regulation of nucleobase−containing compound metabolic process
cellular macromolecule biosynthetic process
mRNA metabolic process
cell cycle process
negative regulation of RNA metabolic process
embryo development
cellular macromolecule catabolic process
DNA metabolic process
regulation of cell cycle
ncRNA metabolic process
central nervous system development
mitotic cell cycle
GeneRatio
p.adjust
Count
c
mESCs
WT/KO: n=4
WT'/CatD: n=3
DMSO/dTAGV-1: n=3
X. laevis
c-MO/m9-MO: n=3
Neural tube stage (DIV10 / NF22)
Neurod4
Syt11
L1cam
Camk2a
Snap23
Stx1b
Syngr2
Synj1
Ppp1r9b
Stmn4
Kif5c
Tub
Gap43
Rab6b
Vamp2
Vps18
normExp (z−scores)
WT
WT'
DMSO
c-MO
KO
CatD
dTAGV-1
m9-MO

Supplementary Fig. 7

Transcriptomic characterisation *X. laevis mettl9*-MO embryos at NF22 stage and comparison with the mouse NPCs lines.

a PCA (left) and volcano plot (right) of differentially expressed genes in *mettl9*-MO embryos and *ctrl*-MO (NF22 RNA-seq). The Y axis of volcano plot represents log10 adjusted (BH-corrected) p-values; two-tailed negative binomial Wald test.
b Top up-regulated Molecular Function GO terms in *mettl9*-MO NF22 embryos. Colour scale shows adjusted p-values (Benjamini-Hochberg correction) of the Hypergeometric test.
c Boxplots showing differentially expressed genes involved in neural processes consistently mis-regulated at DIV10 (mESCs) and NF22 (*X. laevis*). *WT'* is the clonal WT control for $Mettl9^{CatD}$. *c*-MO and *m9*-MO are *ctrl*-MO and *mettl9*-MO, respectively. Error bars show the mean ± SE of N=4 (for $Mettl9^{KO}$ and Ctrl) and N=3 experiments (for $Mettl9^{CatD}$, $Mettl9^{Deg}$ and *X. laevis* and respective Ctrl).

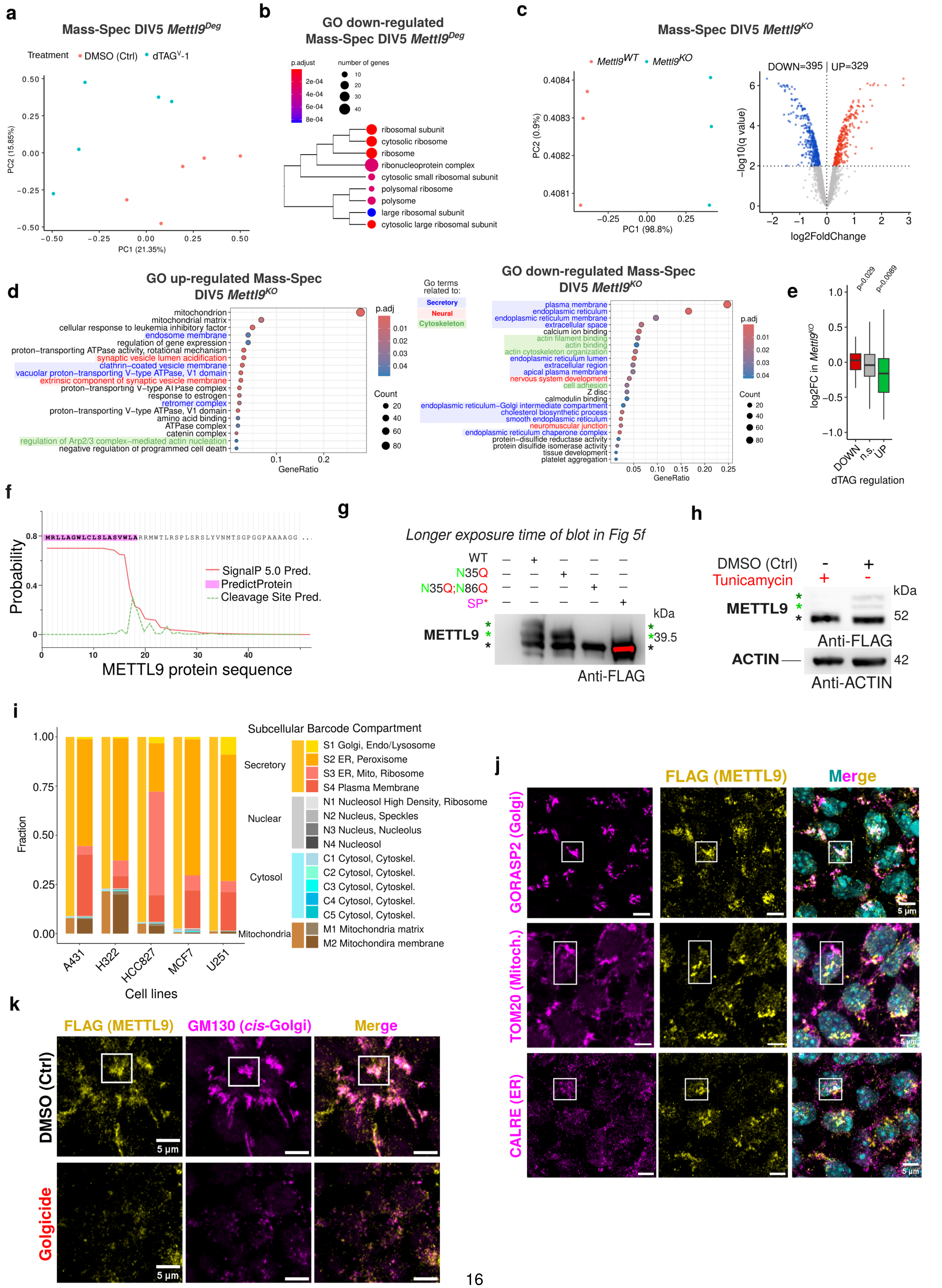
a
Mass-Spec DIV5 Mettl9Deg
Treatment DMSO (Ctrl) dTAGV-1
PC1 (21.35%)
PC2 (15.85%)
b
GO down-regulated Mass-Spec DIV5 Mettl9Deg
p.adjust
number of genes
ribosomal subunit
cytosolic ribosome
ribosome
ribonucleoprotein complex
cytosolic small ribosomal subunit
polysomal ribosome
polysome
large ribosomal subunit
cytosolic large ribosomal subunit
c
Mass-Spec DIV5 Mettl9KO
Mettl9WT Mettl9KO
PC1 (98.8%)
PC2 (0.9%)
DOWN=395 | UP=329
−log10(q value)
log2FoldChange
d
GO up-regulated Mass-Spec DIV5 Mettl9KO
mitochondrion
mitochondrial matrix
cellular response to leukemia inhibitory factor
endosome membrane
regulation of gene expression
proton−transporting ATPase activity, rotational mechanism
synaptic vesicle lumen acidification
clathrin−coated vesicle membrane
vacuolar proton−transporting V−type ATPase, V1 domain
extrinsic component of synaptic vesicle membrane
proton−transporting V−type ATPase complex
response to estrogen
retromer complex
proton−transporting V−type ATPase, V1 domain
amino acid binding
ATPase complex
catenin complex
regulation of Arp2/3 complex−mediated actin nucleation
negative regulation of programmed cell death
GeneRatio
p.adj
Count
Go terms related to:
Secretory
Neural
Cytoskeleton
GO down-regulated Mass-Spec DIV5 Mettl9KO
plasma membrane
endoplasmic reticulum
endoplasmic reticulum membrane
extracellular space
calcium ion binding
actin filament binding
actin binding
actin cytoskeleton organization
endoplasmic reticulum lumen
extracellular region
apical plasma membrane
nervous system development
cell adhesion
Z disc
calmodulin binding
endoplasmic reticulum−Golgi intermediate compartment
cholesterol biosynthetic process
smooth endoplasmic reticulum
neuromuscular junction
endoplasmic reticulum chaperone complex
protein−disulfide reductase activity
protein disulfide isomerase activity
tissue development
platelet aggregation
GeneRatio
e
p=0.029
p=0.0089
log2FC in Mettl9KO
DOWN
n.s.
UP
dTAG regulation
f
MRLLAGWLCLSLASVWLARRMWTLRSPLSRSLYVNMTSGPGGPAAAAGG ...
Probability
SignalP 5.0 Pred.
PredictProtein
Cleavage Site Pred.
METTL9 protein sequence
g
Longer exposure time of blot in Fig 5f
WT
N35Q
N35Q;N86Q
SP*
METTL9
kDa
39.5
Anti-FLAG
h
DMSO (Ctrl)
Tunicamycin
METTL9
kDa
52
Anti-FLAG
ACTIN
42
Anti-ACTIN
i
Subcellular Barcode Compartment
Secretory
Nuclear
Cytosol
Mitochondria
S1 Golgi, Endo/Lysosome
S2 ER, Peroxisome
S3 ER, Mito, Ribosome
S4 Plasma Membrane
N1 Nucleosol High Density, Ribosome
N2 Nucleus, Speckles
N3 Nucleus, Nucleolus
N4 Nucleosol
C1 Cytosol, Cytoskel.
C2 Cytosol, Cytoskel.
C3 Cytosol, Cytoskel.
C4 Cytosol, Cytoskel.
C5 Cytosol, Cytoskel.
M1 Mitochondria matrix
M2 Mitochondira membrane
Fraction
A431
H322
HCC827
MCF7
U251
Cell lines
j
FLAG (METTL9)
Merge
GORASP2 (Golgi)
TOM20 (Mitoch.)
CALRE (ER)
5 µm
k
FLAG (METTL9)
GM130 (cis-Golgi)
Merge
DMSO (Ctrl)
Golgicide
5 µm

Supplementary Fig. 8

Proteomic analysis upon acute or constitutive METTL9 depletion in NSCs and METTL9 subcellular localization in NSCs.

a,b PCA (**a**) and GO analysis (**b**) on proteomic dataset of acutely depleted METTL9-DEG in *Mettl9*^{Deg} NSCs. GO terms (**b**) refer to the down-regulated proteins.
c-d Volcano plot (**c**) and GO analysis (**d**) of the mis-regulated proteins (coloured dots) identified by mass spectrometry in *Mettl9*^{KO} NSCs over *Mettl9*^{WT} (ctrl). Up-regulated genes with an adjusted p value < 0.2 were used for this analysis. The Y axis of volcano plot in (**c**) represents log10 FDR-adjusted p-values; two-tailed moderated t-statistics.
e Boxplot showing the comparison between the mis-regulated proteins of dTAG^{V}-1-treated *Mettl9*^{Deg} NSCs and *Mettl9*^{KO} (Wilcoxon test; down N=58, n.s. N=3630, up N=103). Colour scale in (**b**) and (**d**) shows adjusted p-values (Benjamini-Hochberg correction) of the Hypergeometric test.
f Signal peptide and cleavage sites prediction within METTL9 amino acid sequence, with Signal IP 5.0 and PredictProtein (Almagro Armenteros et al. 2019; Bernhofer et al. 2021).
g Longer exposure of the same WB membrane (Anti-FLAG) as displayed in Fig. 5f highlighting the absence of higher METTL9 bands (green asterisks) in the 3 mutated METTL9 (METTL9-N35Q-FLAG (N35Q), METTL9-N35Q;N86Q-FLAG (N35Q;N86Q), and SP*-METTL9-FLAG (SP*). An Anti-FLAG antibody was used. Black, green and dark green asterisks (*) refer to the lowest, intermediate and top METTL9 bands, respectively. N=3.
h WB showing METTL9-DEG (visualised with an anti-FLAG antibody) in tunicamycin-treated mESCs extracts. An anti-ACTIN antibody was used as a loading control. Asterisks (*) as in (**g**). N=2.
i Relative proportion of METTL9 found in distinct subcellular fractions (each colour refers to a different compartment) in proteomic data from (Orre et al. 2019).
j Representative IF images of *Mettl9*^{Deg} NSCs co-stained with anti-FLAG antibody for METTL9 and: anti-GORASP2 (Golgi) or anti-TOM20 (mitochondria) or anti-CALRE (ER). Nuclei are stained by Hoechst (cyan). Scale bar is 5 μm. IF were performed performed on N=3 differentiation experiments (TOM20 and CALRE) and N=1 (GORASP2).
k Representative IF images of *Mettl9*^{Deg} DMSO- or golgicide-treated NSCs (DIV6), co-stained with anti-FLAG antibody (METTL9) and anti-GM130 (Golgi). Scale bar is 5 μm. Golgicide treatment and IF were performed on N=2 differentiation experiments.

Supplementary Figure 9

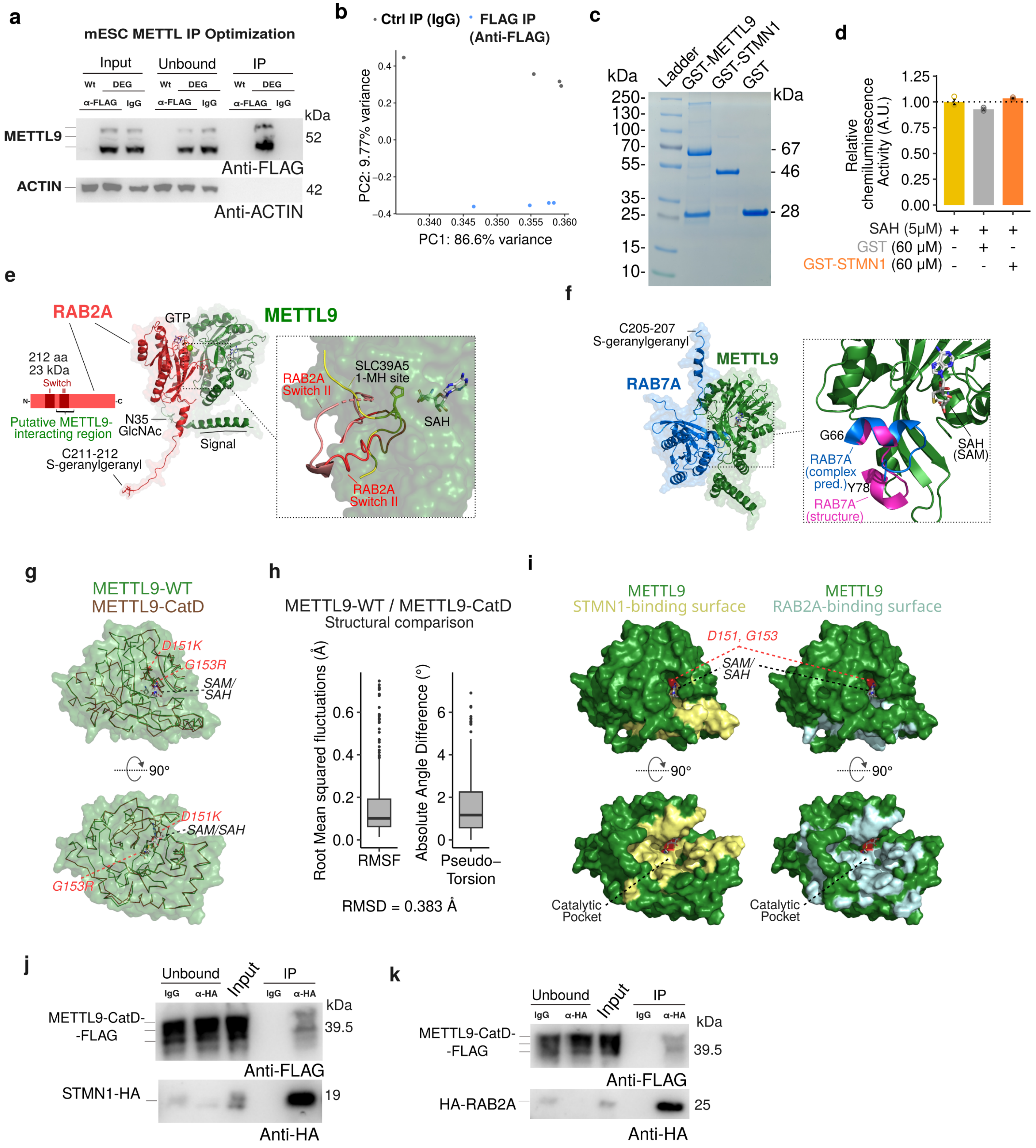

Supplementary Fig. 9
Characterisation of METTL9 protein interactors in mNSCs.

a WB showing a representative METTL9 immunoprecipitation (IP) with anti-FLAG antibody or anti-IgG (Ctrl), from *Mettl9*^{WT} (WT) and *Mettl9*^{Deg} (DEG) mESCs. IP was performed from extracts of N>3 independent differentiation experiments.
b PCA of METTL9 IP-MS samples at DIV4 (NSCs).
c Coomassie-stained poly-acrylamide gel showing the recombinant GST-METTL9 (GST-METTL9-FLAG), GST-STMN1 (GST-STMN1-HA) and GST proteins after affinity purification from *E. coli* extracts. Image is representative of 3 independent elect
rophoresis runs.
d *In vitro* control of the detection method used in methyltransferase assays, showing that the recombinant GST-STMN1 or GST proteins do not interfere with the downstream conversion of S-adenosyl-homocysteine (SAH) into chemiluminescence activity. Concentrations (µM) of each component are shown. Error bars show the mean ± SE of N=3 independent experiments. A.U. is arbitrary unit.
e*,* **f** AlphaFold modelling prediction of RAB2A-METTL9 (**e**) or RAB7-METTL9 (**f**) protein complexes. (RAB2A in red; RAB7 in blue; METTL9 in green).
g Superimposed structures of METTL9-WT (green) and METTL9-CatD (brown backbone), as predicted by AlphaFold.
h Boxplot showing the comparison of the METTL9-WT and METTL9-CatD structures depicted in (**g**), representing the distribution of root mean squared fluctuations and pseudo-torsion angle deviations of the corresponding residues (n=272). The overall root mean square deviation value (RMSD) is shown in the lower part. Points show potential outliers (see Methods).
i Representation of the relative positions of the D151;G153 residues mutated in METTL9-CatD (red) and the predicted interaction surfaces between METTL9 (green) and STMN1 (yellow) or RAB2A (light blue).
j, k WB showing the immunoprecipitation (IP) of STMN1-HA (**j**) or HA-RAB2A (**k**) with anti-HA beads (HA) or IgG (ctrl), after co-expression of STMN1-HA (**j**) or HA-RAB2a (**k**) and METTL9-CatD-FLAG in mESCs (anti-FLAG and anti-HA antibodies). N=2 co-IP (and differentiation) experiments, run in N=4 WB.

Supplementary Figure 10

Codino et al.

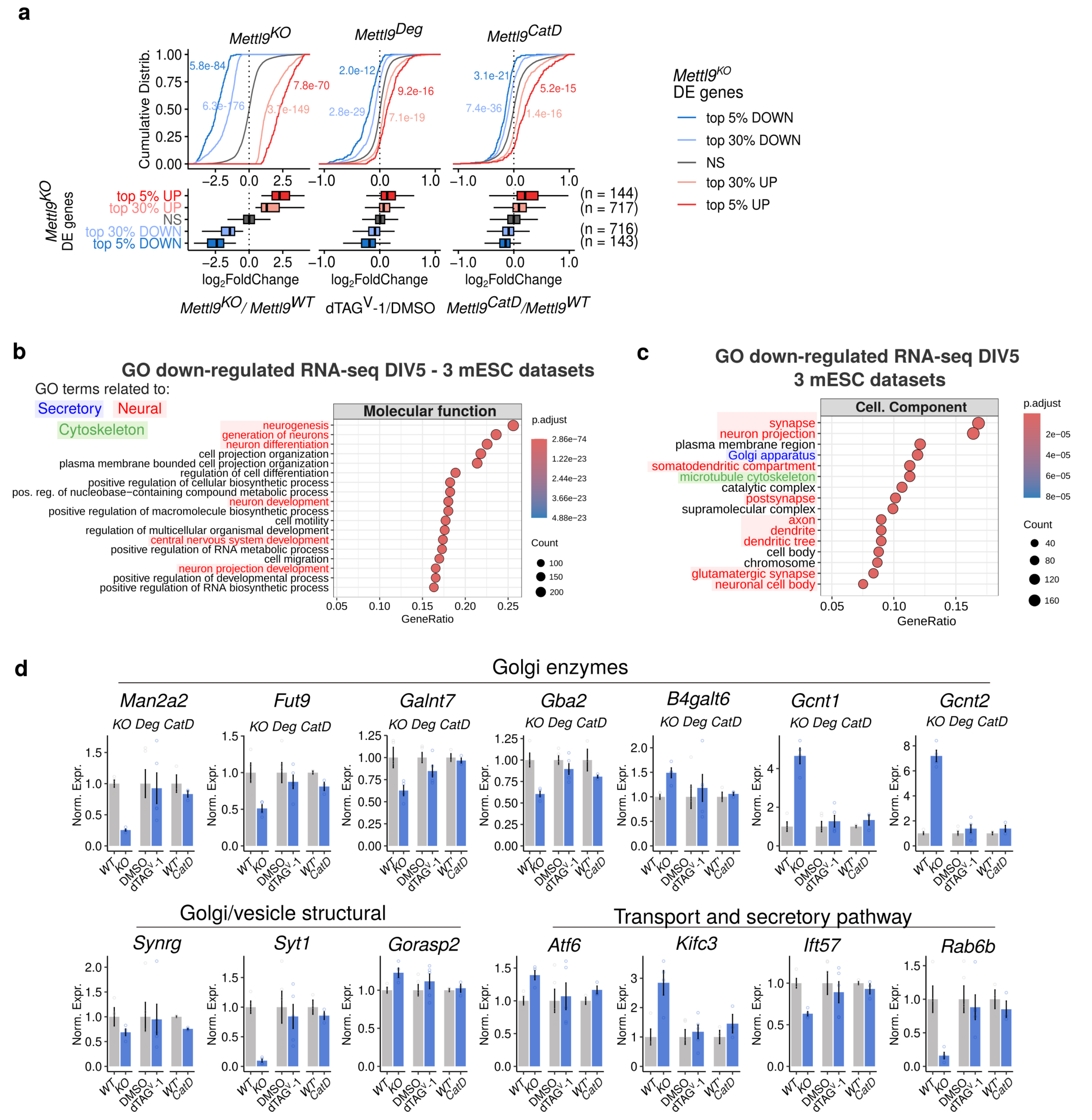

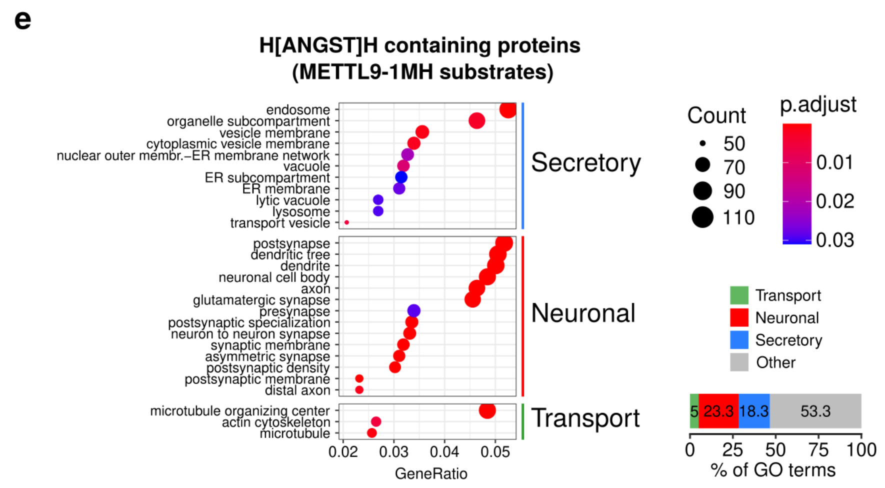

f

METTL9 1MH substrates
(Davydova et al.)

	ER	Cytopl. Vesc.	Golgi
DNAJB12	X		
**MYO18A**		X	X
**MYO1D**		X	
**SLC30A1**	X		X
**SLC30A5**	X	X	X
SLC39A4		X	
SLC39A6	X		
**SLC39A7**	X		X
SLC9A5		X	
STT3B	X		

Supplementary Fig. 10

Biological processes consistently affected across *Mettl9*^{KO}/ ^{CatD}/ ^{Deg} mNSCs and *in silico* analysis of METTL9 substrates.

a Graphs tracking how the genes mis-regulated upon *Mettl9*^{KO} (represented in the four different colors) behave in the other mESC lines under study. The distribution of the (log2) fold changes for each gene group is shown by means of both *cumulative distribution* (top) and *box* plots (bottom). This analysis reveals that up- and down-regulated genes in *Mettl9*^{KO} are generally similarly affected (i.e. they display a concordant, statistically significant up- or down-regulation trend compared to control) also in *Mettl9*^{Deg} and *Mettl9*^{CatD}, albeit to a much smaller extent. For each of the four gene groups considered, the panel also shows its number (on the right of the corresponding boxplot line). P values from the Kolmogorov-Smirnov test are superimposed on the respective cumulative distributions.
b, c Most enriched Molecular Function (**b**) and Cellular Component (**c**) GO terms for the significantly down-regulated genes across the three mouse cell lines.
d Normalised transcript per million expression of a subset of differentially expressed genes encoding for Golgi enzymes; structural Golgi and vesicles' proteins and transport/secretory pathway-related proteins in *Mettl9*^{KO}, dTAG^{V}-1-treated *Mettl9*^{Deg} and *Mettl9*^{CatD} lines and their relative controls (DIV5). Error bars show the mean ± SE of N=4, N=5 and N=3 experiments (for *Mettl9*^{KO}, *Mettl9*^{Deg} and *Mettl9*^{CatD}, respectively, and relative Ctrls).
e Some of the most enriched GO terms (Transport, Endomembrane and Neuronal) in H[ANGST]H-containing mouse proteins (i.e. potential METTL9 substrates). Colour scale in (**b**), (**c**) and (**e**) shows adjusted p-values (Benjamini-Hochberg correction) of the Hypergeometric test.
f METTL9-1MH substrates, experimentally validated by Davydova et al. (Davydova et al. 2021), whose localisation is within ER, Cytoplasmic Vesicles (i.e. endosomes, secretory/synaptic vesicles) and Golgi.

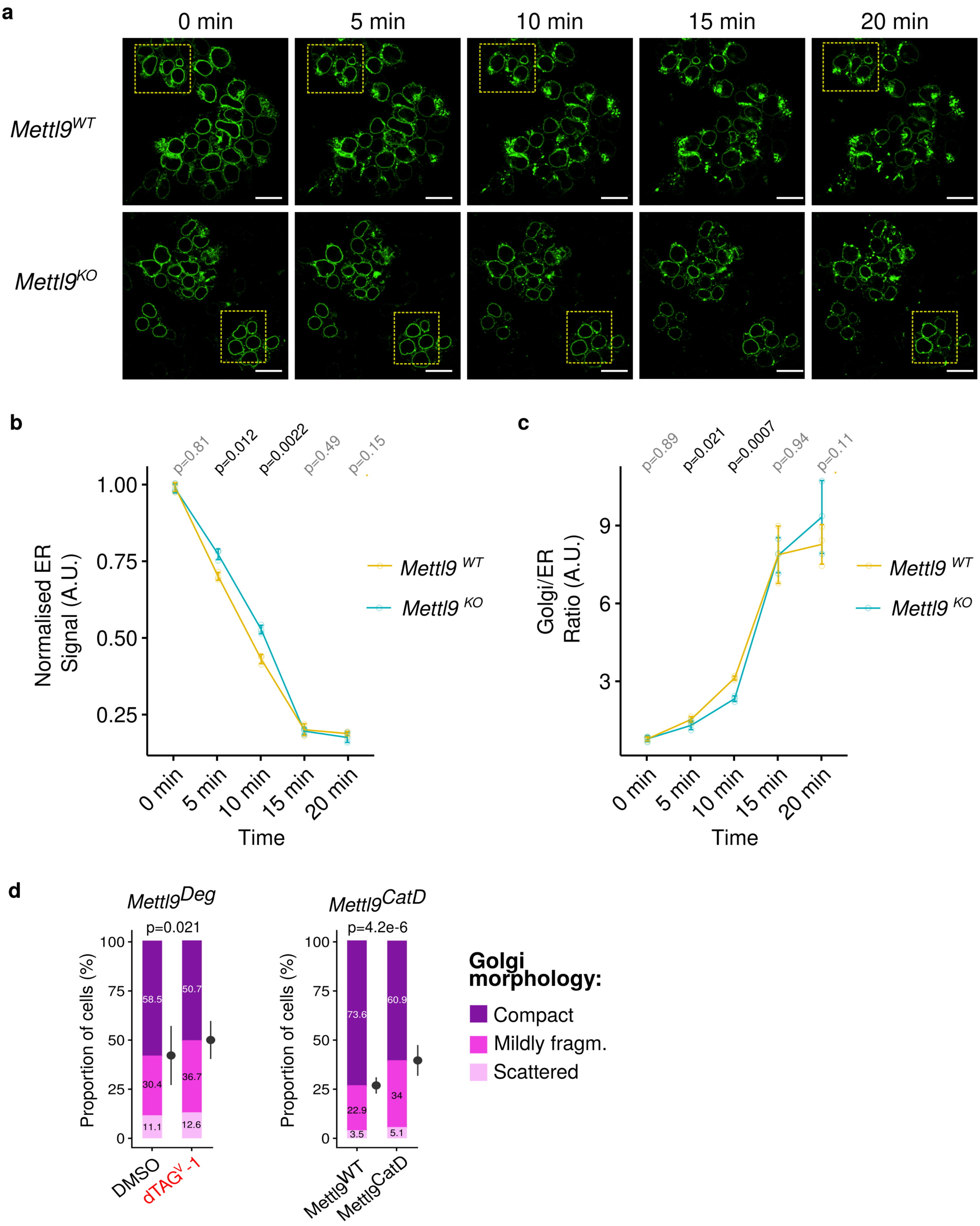
a
0 min
5 min
10 min
15 min
20 min
Mettl9WT
Mettl9KO
b
p=0.81
p=0.012
p=0.0022
p=0.49
p=0.15
Normalised ER Signal (A.U.)
Time
Mettl9 WT
Mettl9 KO
c
p=0.89
p=0.021
p=0.0007
p=0.94
p=0.11
Golgi/ER Ratio (A.U.)
Time
d
Mettl9Deg
p=0.021
Mettl9CatD
p=4.2e-6
Proportion of cells (%)
DMSO
dTAGV-1
Mettl9WT
Mettl9CatD
Golgi morphology:
Compact
Mildly fragm.
Scattered

Supplementary Fig. 11

Cellular trafficking analysis by RUSH in *Mettl9^{KO}* NSCs and quantification of Golgi morphology in *Mettl9^{Deg}* and *Mettl9^{CatD}*.

a Entire fields of view of the corresponding zoomed-in images displayed in Fig.8b, showing ManII-SBP-EGFP signal in *Mettl9^{WT}* and *Mettl9^{KO}* NSCs, at 0, 5, 10, 15 and 20 minutes after Biotin addition. Scale bar is 20 µm.
b, c Normalised ManII-SBP-EGFP signal in the ER (**b**) and Golgi over ER signal ratio (**c**) in *Mettl9^{WT}* and *Mettl9^{KO}* NSCs across 3 independent RUSH experiments. Paired t-test p values are shown above each time point; error bars represent mean ± SE of N=3 independent experiments.
d Relative number of cells displaying a compact, mildly fragmented or scattered Golgi in dTAG^{V}-1-treated *Mettl9^{Deg}* compared to DMSO-treated NSCs and in *Mettl9^{CatD}* or *Mettl9^{WT}* mNSCs. Error bars represent mean ± SD; number of cells counted are above each panel (χ2 test). N=2 IF and differentiation experiment for each condition.

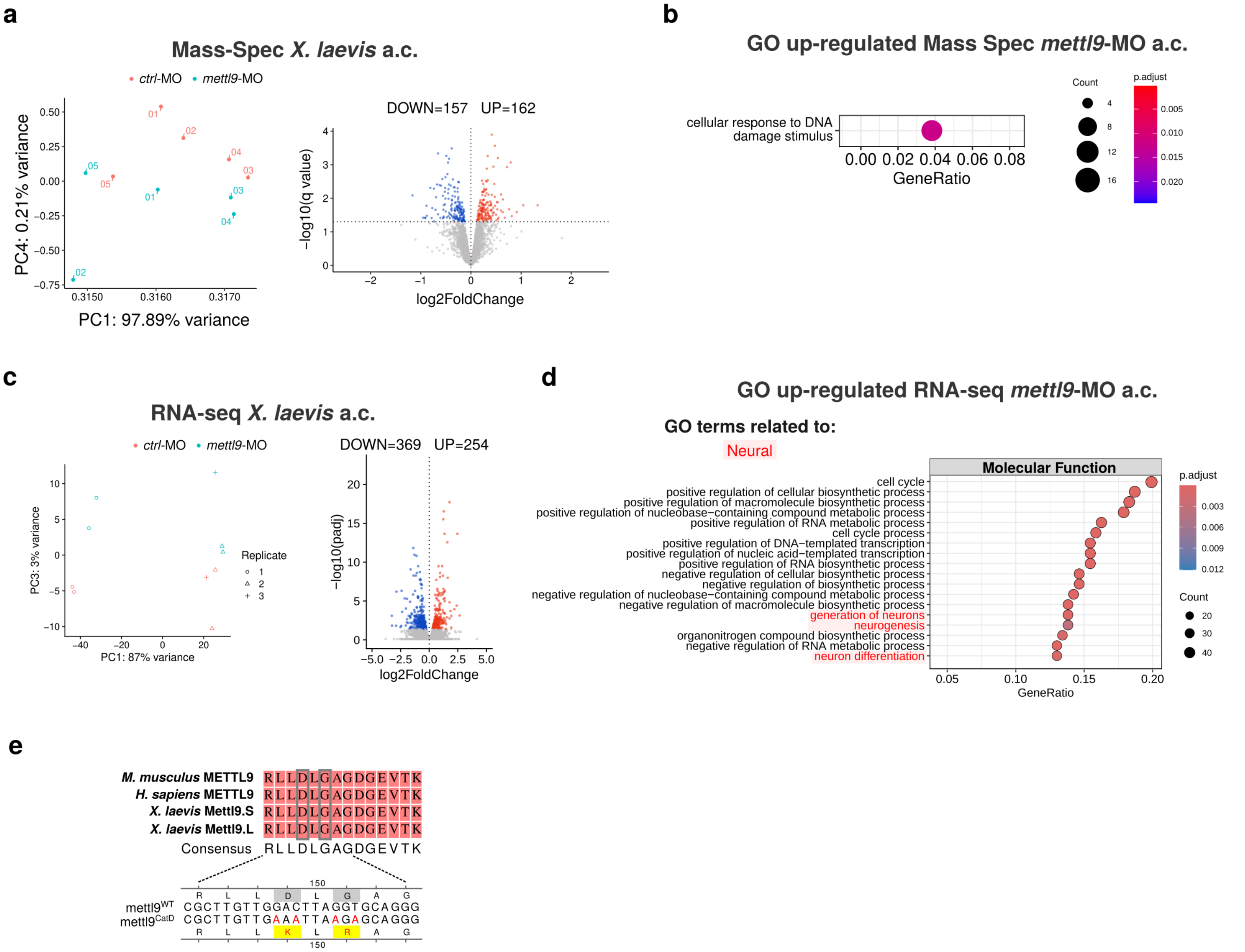
a
Mass-Spec X. laevis a.c.
ctrl-MO mettl9-MO
PC4: 0.21% variance
PC1: 97.89% variance
DOWN=157 UP=162
−log10(q value)
log2FoldChange
b
GO up-regulated Mass Spec mettl9-MO a.c.
cellular response to DNA damage stimulus
GeneRatio
Count
p.adjust
c
RNA-seq X. laevis a.c.
ctrl-MO mettl9-MO
PC3: 3% variance
PC1: 87% variance
Replicate
DOWN=369 UP=254
−log10(padj)
log2FoldChange
d
GO up-regulated RNA-seq mettl9-MO a.c.
GO terms related to:
Neural
Molecular Function
cell cycle
positive regulation of cellular biosynthetic process
positive regulation of macromolecule biosynthetic process
positive regulation of nucleobase−containing compound metabolic process
positive regulation of RNA biosynthetic process
cell cycle process
positive regulation of DNA−templated transcription
positive regulation of nucleic acid−templated transcription
positive regulation of RNA biosynthetic process
negative regulation of cellular biosynthetic process
negative regulation of biosynthetic process
negative regulation of nucleobase−containing compound metabolic process
negative regulation of macromolecule biosynthetic process
generation of neurons
neurogenesis
organonitrogen compound biosynthetic process
negative regulation of RNA metabolic process
neuron differentiation
GeneRatio
p.adjust
Count
e
M. musculus METTL9 RLLDLGAGDGEVTK
H. sapiens METTL9 RLLDLGAGDGEVTK
X. laevis Mettl9.S RLLDLGAGDGEVTK
X. laevis Mettl9.L RLLDLGAGDGEVTK
Consensus RLLDLGAGDGEVTK
mettl9WT CGCTTGTTGGACTTAGGTGCAGGG
mettl9CatD CGCTTGTTGAAATTAAGAGCAGGG

Supplementary Fig. 12

Proteomic and transcriptomic analysis of *Mettl9*-depleted *X. laevis* animal caps (a.c.) and design of *mettl9*^{CatD} mRNA for the rescue.

a PCA (left) and volcano (right) plots of mis-regulated proteins in *mettl9*-MO neuralised animal caps (a.c.) vs. *ctrl*-MO neuralised a.c. The Y axis of volcano plot represents log10 FDR-adjusted p-values; two-tailed moderated t-statistics.
b Up-regulated GO term in the *mettl*-MO proteome versus *ctrl*-MO proteome of neuralised a.c. (see Methods).
c PCA (left) and volcano (right) plots of differentially expressed genes in the *mettl9*-MO versus control neuralised animal caps RNA-seq experiment. The Y axis of volcano plot represents log10 adjusted (BH-corrected) p-values; two-tailed negative binomial Wald test.
d Most up-regulated Molecular Function GO terms of DEGs in the same experiment described in (**c**). Colour scale in (**b**) and (**d**) shows adjusted p-values (Benjamini-Hochberg correction) of the Hypergeometric test.
e Schematic showing the 4 nucleotides mutagenised in *mettl9*^{CatD} mRNA encoding D149K and G151R within the conserved amino acid sequence of *X. laevis* Mettl9 catalytic domain.

Displayed images.

Uncropped Supplemental Figure 4e

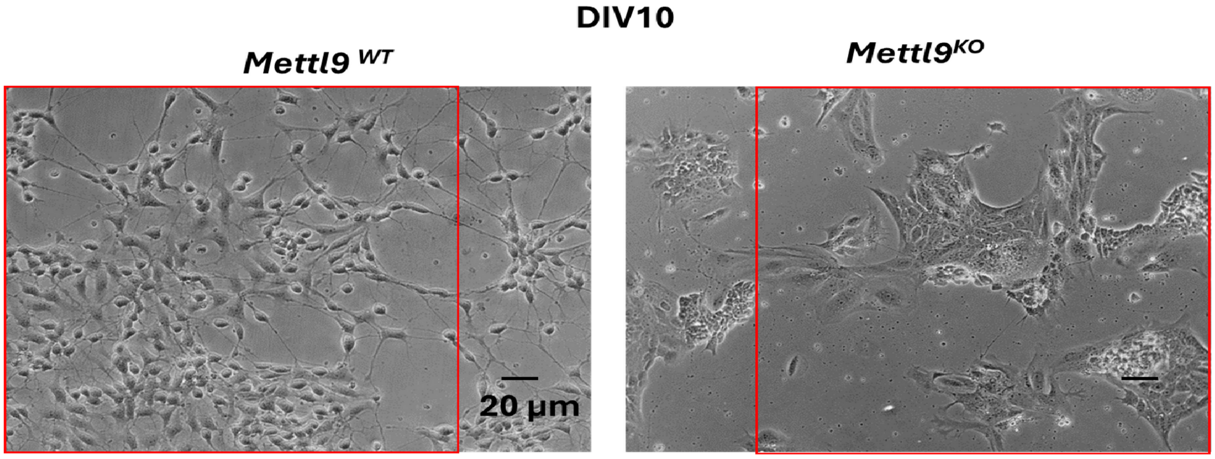

Uncropped Supplemental Figure 5a

Displayed in Fig

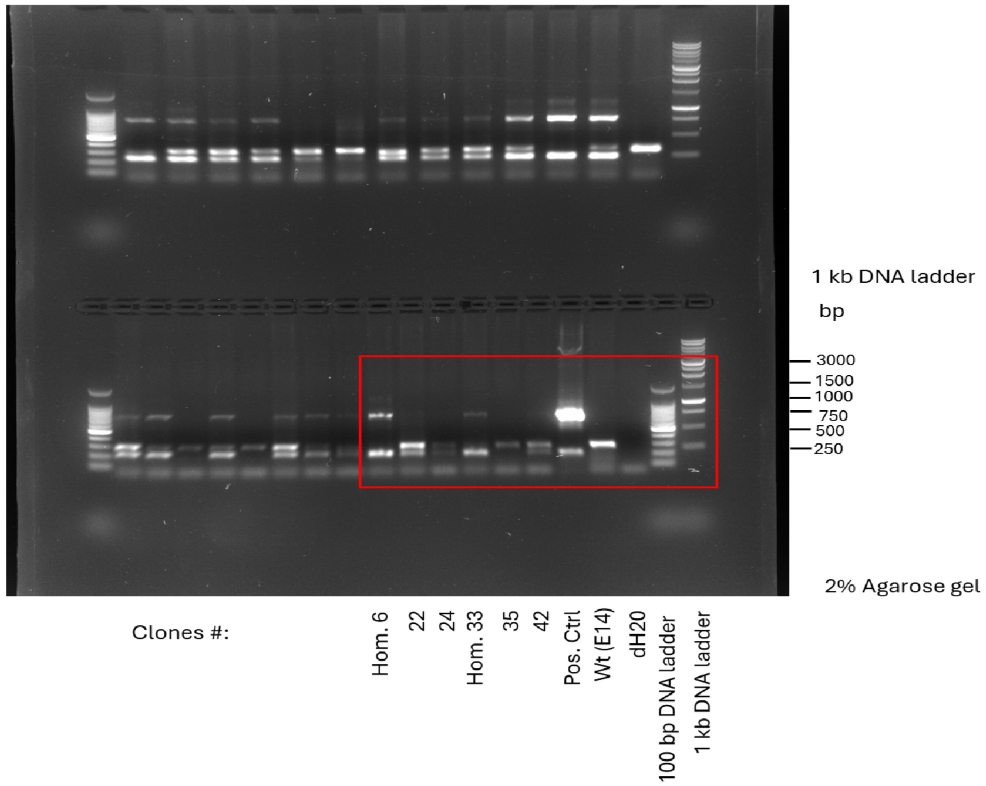

Uncropped Supplemental Figure 5f

Displayed in Fig

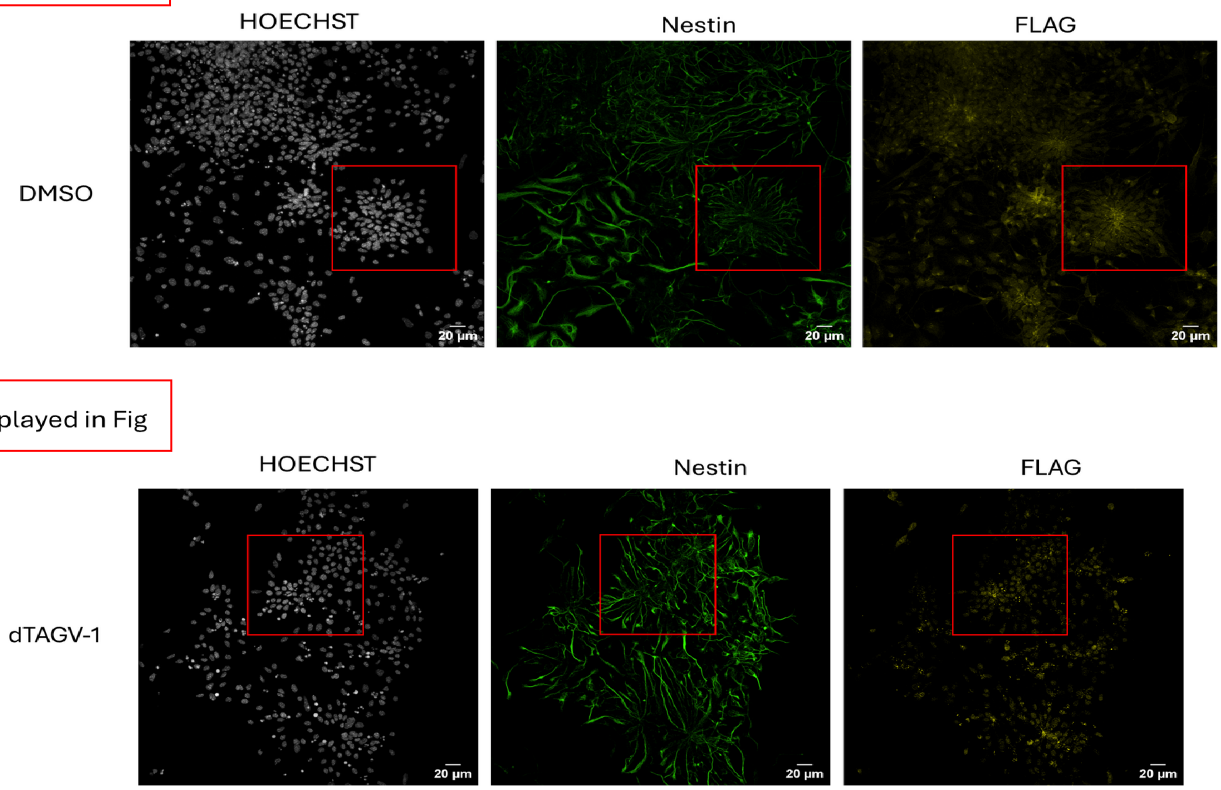

Uncropped Supplemental Figure 6a

Displayed in Fig

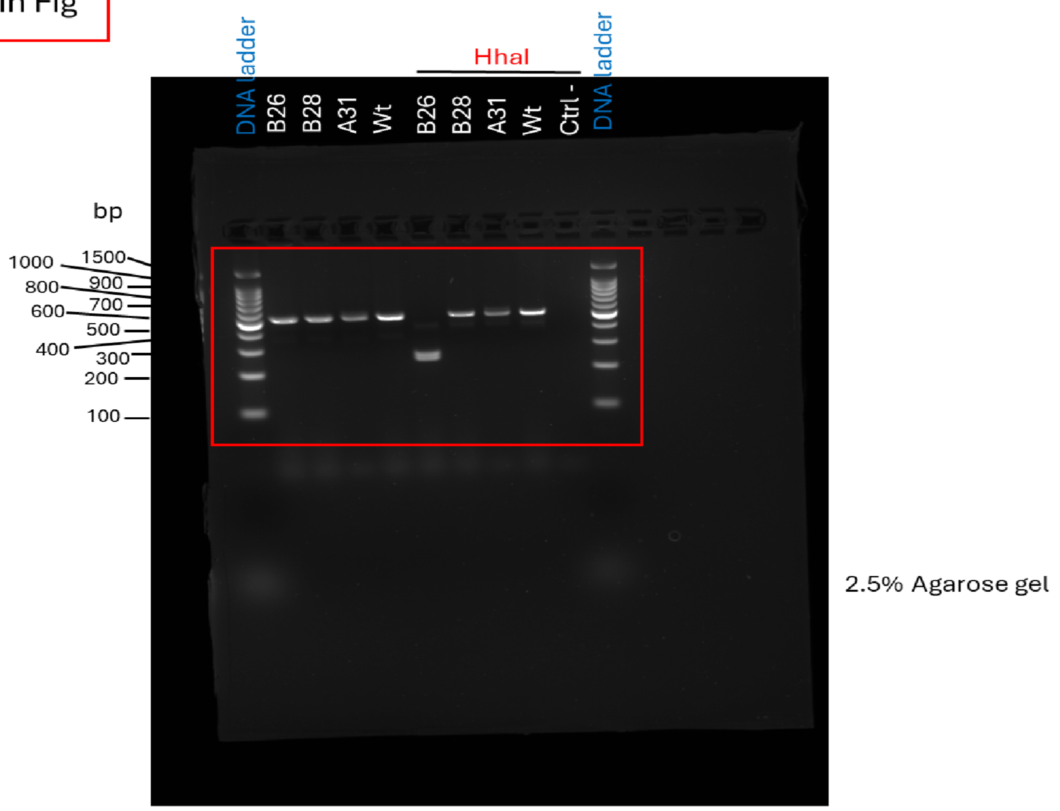

Uncropped Supplemental Figure 6b

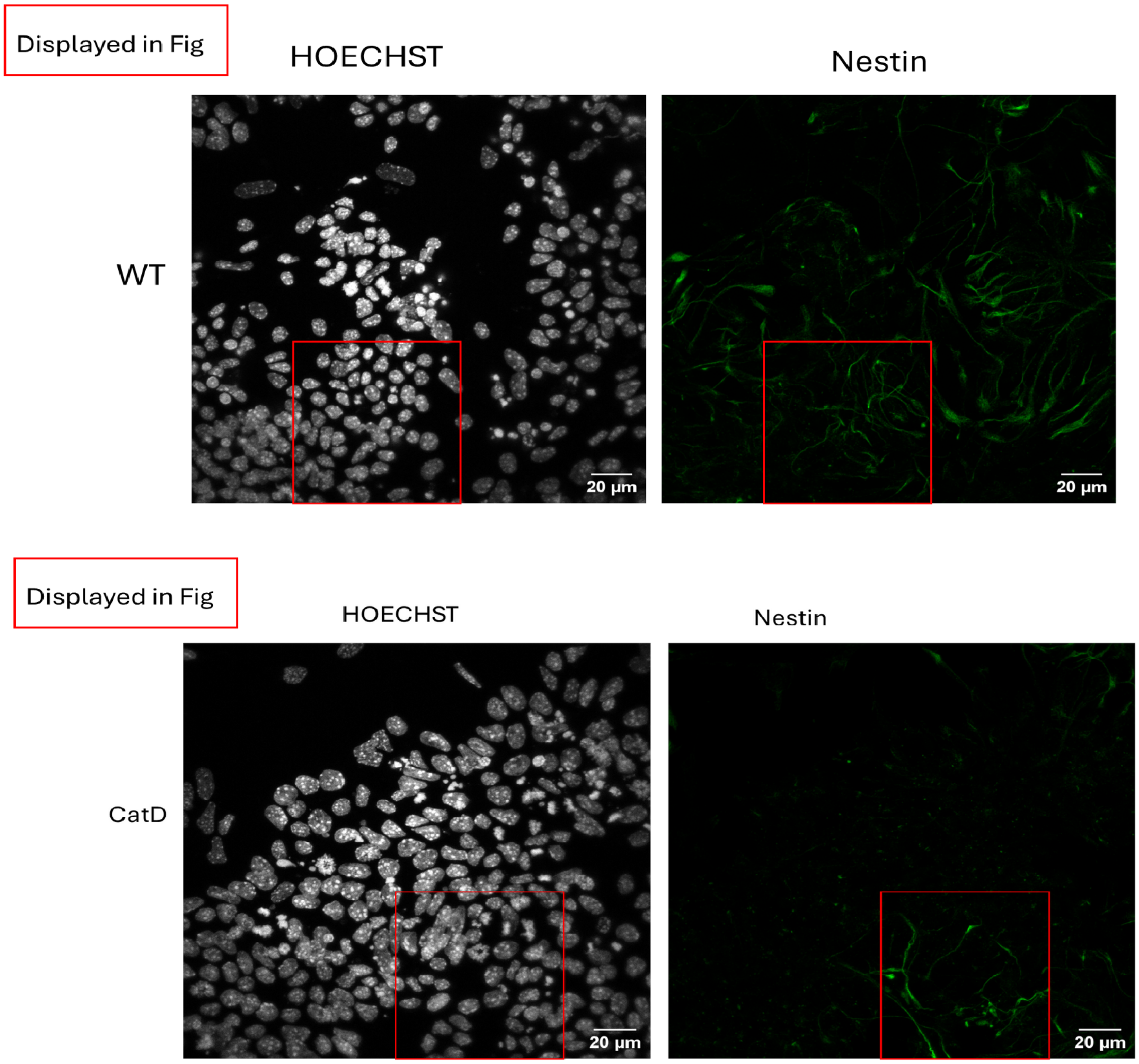

Uncropped Supplemental Figure 8g

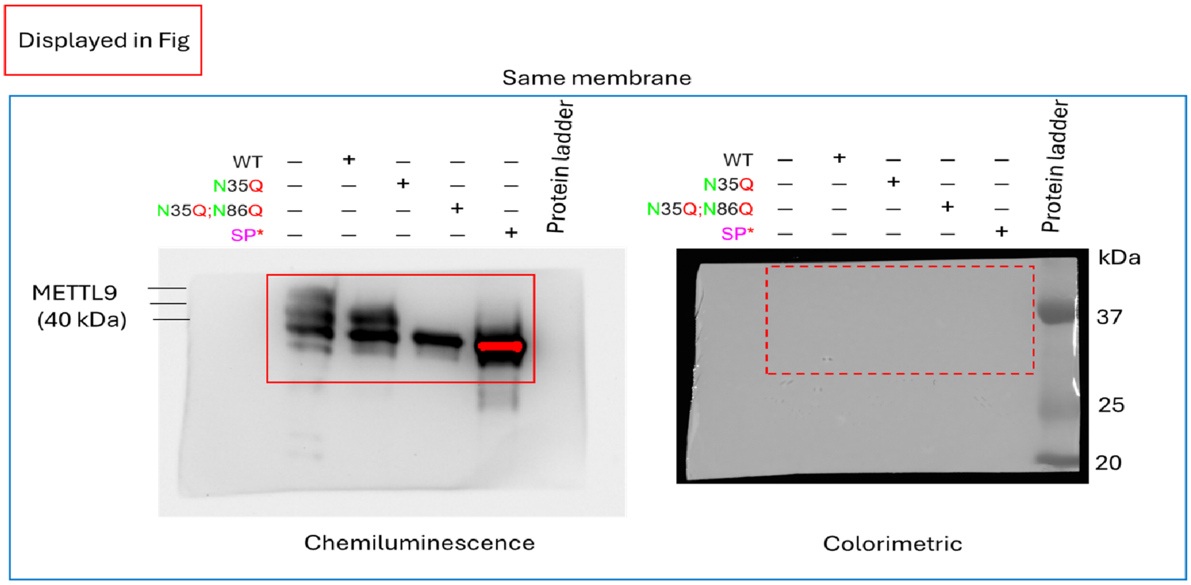

Uncropped Supplemental Figure 8h

Displayed in Fig

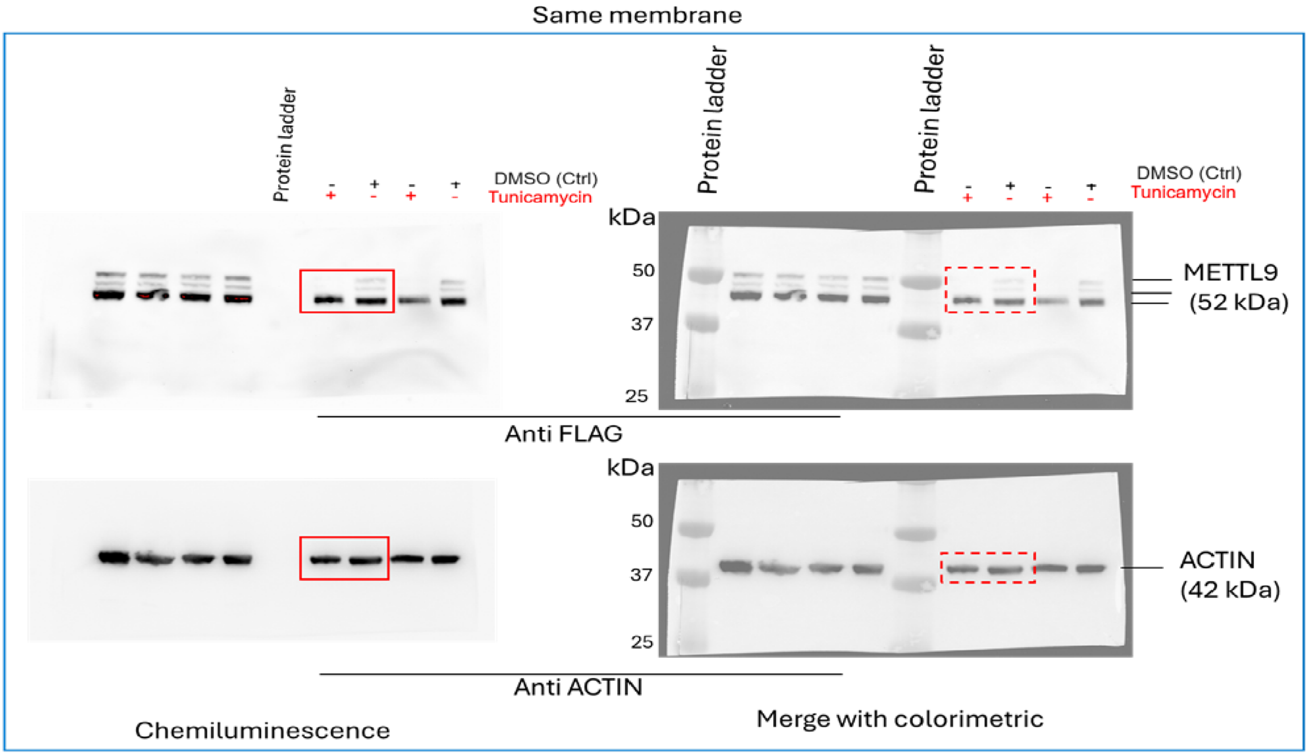

Uncropped Supplemental Figure 8k

Displayed in Fig

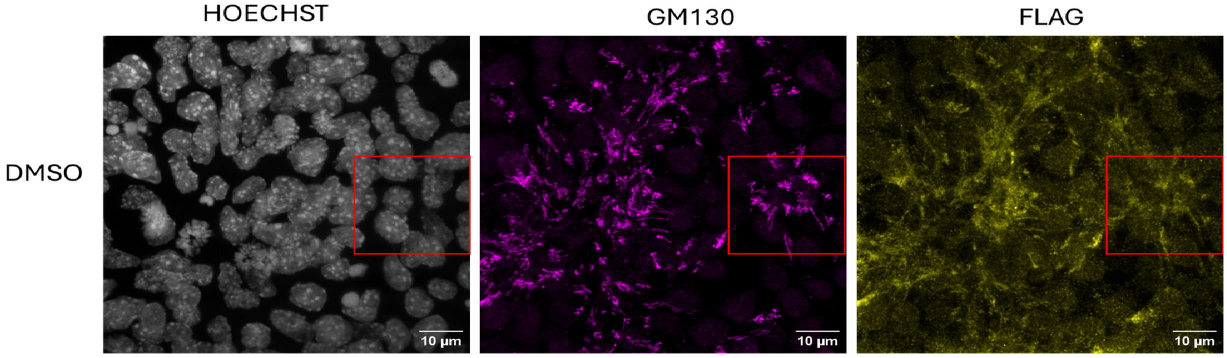

Displayed in Fig

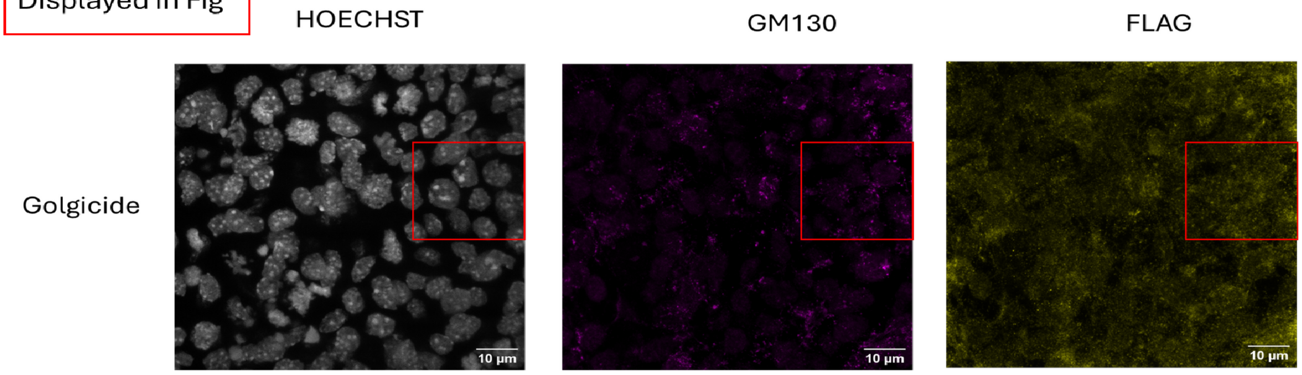

Uncropped Supplemental Figure 8j

Displayed in Fig

GORASP2 FLAG HOECHST

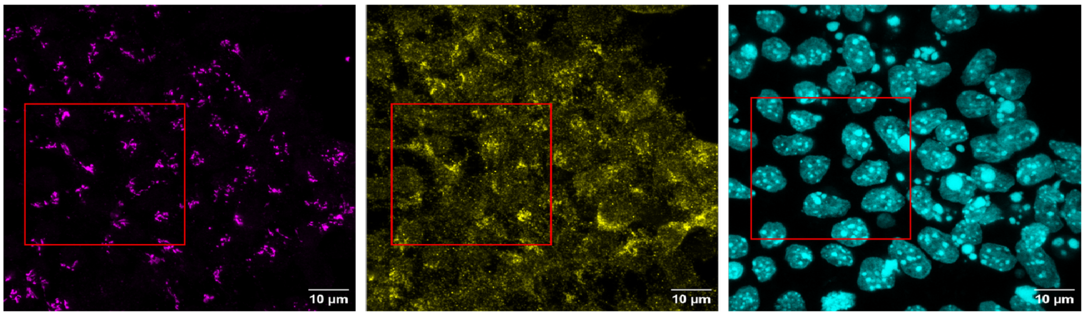

Displayed in Fig.

TOM20 FLAG HOECHST

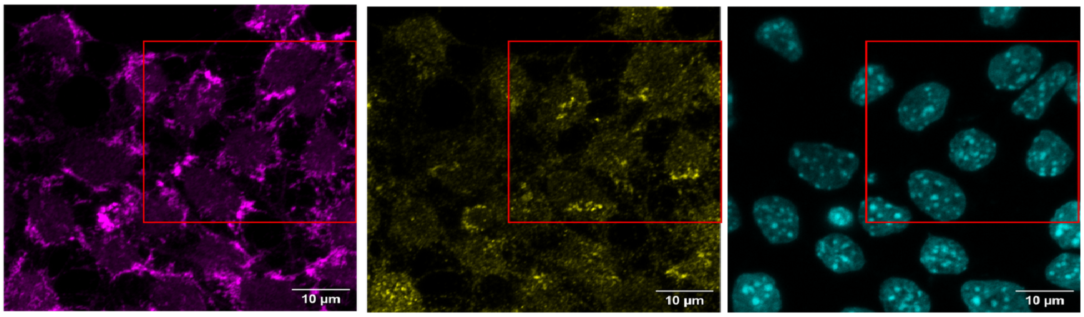

Displayed in Fig.

CALRE FLAG HOECHST

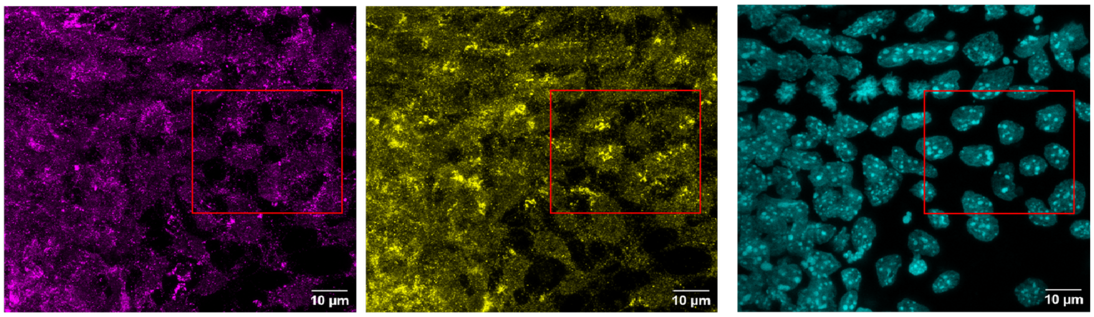

Uncropped Supplemental Figure 9a

Displayed in Fig

Same membrane

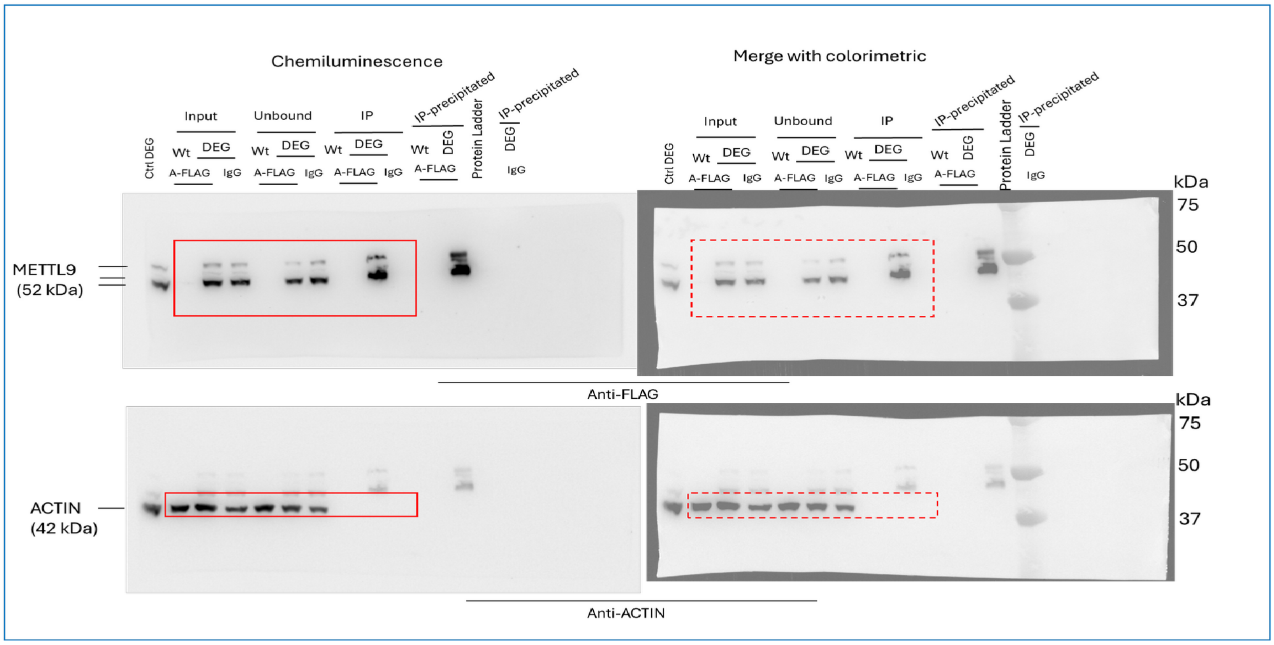

Uncropped Supplemental Figure 9c

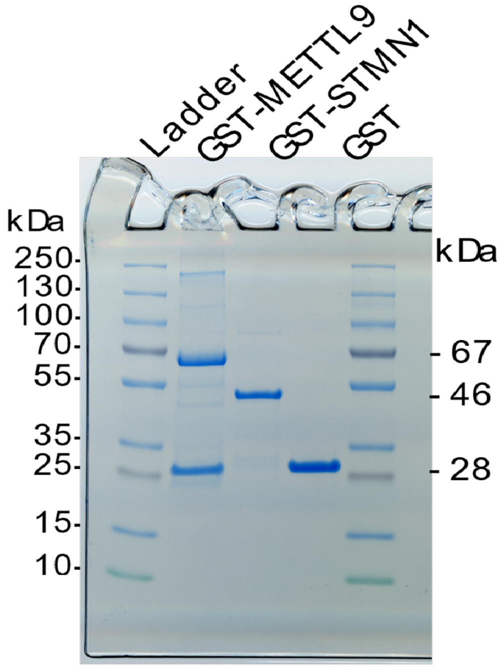

Uncropped Supplemental Figure 9j

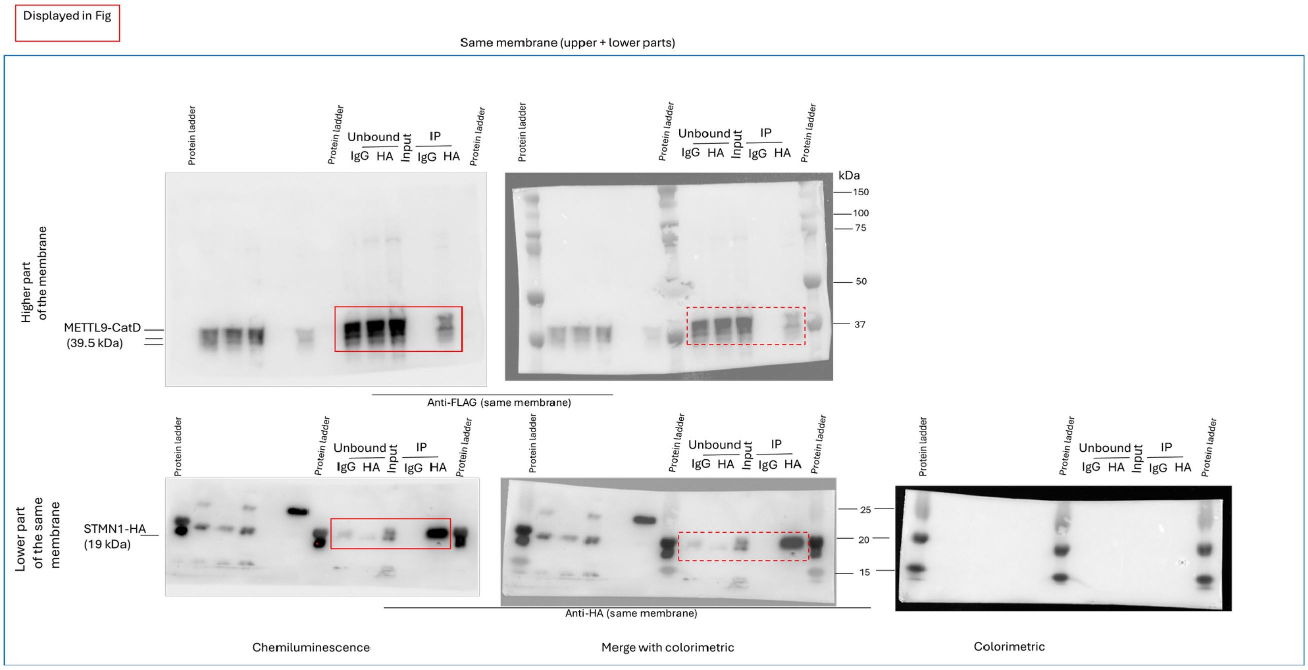

Uncropped Supplemental Figure 9k

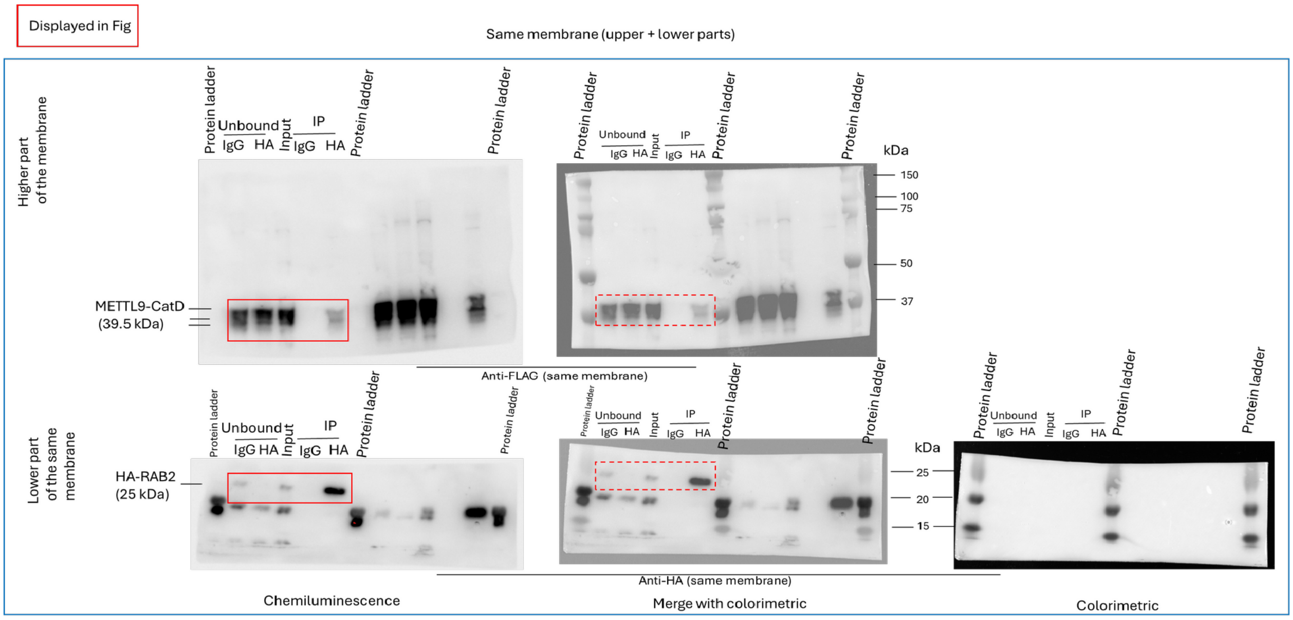

Supplement: Supplementary file 1 — Supplementary Information [file 41467_2025_62414_MOESM1_ESM.pdf]
