# Supplementary material for: METTL9 sustains vertebrate neural development primarily via non-catalytic functions
Source: Nat Commun. 2025 Aug 1;16:7051. doi: 10.1038/s41467-025-62414-5 (PMC12313917; doi:10.1038/s41467-025-62414-5)
Supplement: Supplementary file 2 — Description of Additional Supplementary Files [file 41467_2025_62414_MOESM2_ESM.pdf]

Description of Additional Supplementary Files

Supplementary Data 1, related to Fig. 2-4,8 and Supplementary Fig. 4-7,10,12.

Differential gene expression analysis results for all the experimental conditions and time points assayed by RNA sequencing. Each table includes mean normalised pseudocounts, log2 fold changes, p values of the two-tailed negative binomial Wald test and their corresponding Benjamini-Hochberg adjusted p values.

Supplementary Data 2, related to Fig. 2-4,8 and Supplementary Fig. 4-7,10,12.

Significantly enriched Gene Ontology analyses for the differentially expressed genes as detected by RNA sequencing. Each table includes the ratio of genes from the input gene list present in the gene set, the ratio of background genes that are annotated to the gene set, raw p values from the hypergeometric test, Benjamini-Hochberg and FDR corrected p values and all the input genes contained within the corresponding set.

Supplementary Data 3, related to Fig. 5,6,8 and Supplementary Fig. 8,9,12.

Mass spectrometry results, including total proteomics and immunoprecipitation followed by mass spectrometry. Each table includes log2 fold changes (log2 Enrichments for the IP-MS experiment), p values of the two-tailed (one-tailed for the IP-MS experiment) moderated t-statistics and their corresponding FDR-adjusted values.

Supplementary Data 4, related to Fig. 1-8 and Supplementary Fig. 2-6,8,9,11.

Oligonucleotides used in the study.

Supplementary Data 5, related to Fig. 2-6,8 and Supplementary Fig. 4-10,12.

Experimental samples and layout of the transcriptomic and proteomics experiments, including sample numerosity and experimental batches.

Supplementary Data 6, related to Fig. 6 and Supplementary Fig. 2 and 9.

Pymol sessions representing the molecular structures predicted by AlphaFold for METTL9-STMN1, METTL9-RAB2A and METTL9-RAB7A dimers, and for Xenopus and mouse or WT and CatD METTL9 structural comparisons (superimposed).
