# Supplementary material for: METTL9 sustains vertebrate neural development primarily via non-catalytic functions
Source: Nat Commun. 2025 Aug 1;16:7051. doi: 10.1038/s41467-025-62414-5 (PMC12313917; doi:10.1038/s41467-025-62414-5)
Supplement: Supplementary file 9 — Reporting Summary [file 41467_2025_62414_MOESM9_ESM.pdf]

nature portfolio

Corresponding author(s): Luca Pandolfini

Last updated by author(s): June 19th, 2025

Reporting Summary

Nature Portfolio wishes to improve the reproducibility of the work that we publish. This form provides structure for consistency and transparency in reporting. For further information on Nature Portfolio policies, see our Editorial Policies and the Editorial Policy Checklist.

Statistics

For all statistical analyses, confirm that the following items are present in the figure legend, table legend, main text, or Methods section.

n/a	Confirmed	
☐	☒	The exact sample size (*n*) for each experimental group/condition, given as a discrete number and unit of measurement
☐	☒	A statement on whether measurements were taken from distinct samples or whether the same sample was measured repeatedly
☐	☒	The statistical test(s) used AND whether they are one- or two-sided<br>*Only common tests should be described solely by name; describe more complex techniques in the Methods section.*
☒	☐	A description of all covariates tested
☐	☒	A description of any assumptions or corrections, such as tests of normality and adjustment for multiple comparisons
☐	☒	A full description of the statistical parameters including central tendency (e.g. means) or other basic estimates (e.g. regression coefficient) AND variation (e.g. standard deviation) or associated estimates of uncertainty (e.g. confidence intervals)
☐	☒	For null hypothesis testing, the test statistic (e.g. *F*, *t*, *r*) with confidence intervals, effect sizes, degrees of freedom and *P* value noted<br>*Give P values as exact values whenever suitable.*
☒	☐	For Bayesian analysis, information on the choice of priors and Markov chain Monte Carlo settings
☒	☐	For hierarchical and complex designs, identification of the appropriate level for tests and full reporting of outcomes
☐	☒	Estimates of effect sizes (e.g. Cohen's *d*, Pearson's *r*), indicating how they were calculated

Our web collection on statistics for biologists contains articles on many of the points above.

Software and code

Policy information about availability of computer code

Data collection	NIS-Elements (Nikon) was used for the acquisition of confocal images.
Data analysis	CHOPCHOP (https://chopchop.cbu.uib.no/); minimap2 (2.26); Integrative Genome Viewer (2.18.2); Cas-OFFinder (github.com/snugel/cas-offinder); DeepVariant (1.6.0); glNexus (1.4.1); Sniffles2 (2.2); vcftools (0.1.15); bedtools (2.29.2); bcftools (1.21); CRISPOR (https://crispor.gi.ucsc.edu/); Fiji (2.14.0); MaxQuant (2.1.2.0); Perseus (multiple vv.); Salmon(1.8.0); NetNGlyc (1.0); AlphaFold (2.3.2); SnapGene (multiple vv.); GNU Image Manipulation Program (GIMP 2.10.36); The R statistical environment (4.3.0 to 4.4.1) and multiple associated CRAN/Bioconductor packages (DEqMS, pair, lmFit, DESeq2, clusterProfiler, bio3d, tidyverse, cowplot, enrichplot).

For manuscripts utilizing custom algorithms or software that are central to the research but not yet described in published literature, software must be made available to editors and reviewers. We strongly encourage code deposition in a community repository (e.g. GitHub). See the Nature Portfolio guidelines for submitting code & software for further information.

Data

Policy information about availability of data

All manuscripts must include a data availability statement. This statement should provide the following information, where applicable:

- Accession codes, unique identifiers, or web links for publicly available datasets
- A description of any restrictions on data availability
- For clinical datasets or third party data, please ensure that the statement adheres to our policy

Raw RNA sequencing data from mESCs and X. laevis experiments were submitted to the SRA archive with project identifiers PRJNA1111296 and PRJNA1111433, respectively.
Long-read whole genome sequences of the parental cell line and MettlKO clones #88 and #90 are deposited under SRA project PRJNA1242812.
The mass spectrometry proteomics data (both raw and protein groups tables) have been deposited to the ProteomeXchange Consortium187 via the PRIDE partner repository with the dataset identifier PXD053437.

Research involving human participants, their data, or biological material

Policy information about studies with human participants or human data. See also policy information about sex, gender (identity/presentation), and sexual orientation and race, ethnicity and racism.

Reporting on sex and gender	Sex determination of the Xenopus embryos used in this study was not conducted due to the technical constraints associated with sex identification at early developmental stages; however, this limitation is not expected to impact the results, as the large sample sizes employed minimize potential sex-related variability.<br><br>The genetic sex composition of the 6 patients (described in the Decipher project) was of 2 females (327478,276830) and 4 males (276011, 412447, 266291, 322729).
Reporting on race, ethnicity, or other socially relevant groupings	N/A
Population characteristics	N/A
Recruitment	N/A
Ethics oversight	N/A

Note that full information on the approval of the study protocol must also be provided in the manuscript.

Field-specific reporting

Please select the one below that is the best fit for your research. If you are not sure, read the appropriate sections before making your selection.

☒ Life sciences ☐ Behavioural & social sciences ☐ Ecological, evolutionary & environmental sciences

For a reference copy of the document with all sections, see nature.com/documents/nr-reporting-summary-flat.pdf

Life sciences study design

All studies must disclose on these points even when the disclosure is negative.

Sample size	Sample sizes were decided taking into account the experimental layouts described in the relevant literature. No sample size calculation was performed.
Data exclusions	No data were excluded
Replication	The finding described in the work were all successfully confimed by replication for at least 3 independent experiments, which have been collectively included, analysed and reported in the statistics
Randomization	N/A
Blinding	The labels of single images or single temporal series were automatically scrambled before any manual cell counting, morphology classification and cell mask selection (for RUSH analysis), to avoid any bias.<br>For Xenopus phenotype classification, the labels of each individual test tube containing a batch of embryos were scrambled prior to manual microscopical evaluation.

Reporting for specific materials, systems and methods

We require information from authors about some types of materials, experimental systems and methods used in many studies. Here, indicate whether each material, system or method listed is relevant to your study. If you are not sure if a list item applies to your research, read the appropriate section before selecting a response.

Materials & experimental systems

n/a	Involved in the study	
☐	☒	Antibodies
☐	☒	Eukaryotic cell lines
☒	☐	Palaeontology and archaeology
☐	☒	Animals and other organisms
☒	☐	Clinical data
☒	☐	Dual use research of concern
☒	☐	Plants

Methods

n/a	Involved in the study	
☒	☐	ChIP-seq
☒	☐	Flow cytometry
☒	☐	MRI-based neuroimaging

Antibodies

Antibodies used

Immuno-precipitation:
anti-FLAG antibody (Sigma Aldrich, #F1804); normal mouse IgG (Santa Cruz Biotechnology, #sc-2025); Pierce™ Anti-HA Magnetic Beads (Thermo Fisher Scientific, #88836).

Western Blot:
[Primary] mouse anti-FLAG (Merck Millipore, #F3165), rabbit anti-METTL9 (Custom antibody, Biomatik LLC), rabbit anti-HA (BioLegend, #902301), rabbit anti-Alpha Tubulin antibody (Proteintech, #11224-1-AP), anti-β-Actin–Peroxidase Mouse monoclonal (Merck Millipore, #A3854)
[Secondary] HRP-conjugated Goat anti-Mouse IgG (Proteintech, #SA00001-1), HRP-conjugated Goat anti-Rabbit IgG (Thermo Fisher Scientific, #G-21234), (1:10000).

Immunofluorescence
[Primary] anti-FLAG (Sigma, #F1804), anti-GOLGA2/GM130 (Proteintech, #11308-1-AP), anti-NESTIN (Cell Signaling Technology, E4O9E XP® BK73349S), anti-TOM20 (Proteintech, #11802-1-AP), anti-GORASP2 (Proteintech, #10598-1-AP), anti-CALRETICULIN (Proteintech, #27298-1-AP), anti-FLAG (Sigma, #F1804), rabbit anti-HA-Tag (C29F4, Cell Signaling, #3724).
[Secondary] Goat anti-Mouse Alexa Fluor™ Plus 488 (Thermo Fisher Scientific, #A32723TR), Goat anti-Rabbit Alexa Fluor™ Plus 488 (Thermo Fisher Scientific, #A32731TR), Goat anti-Mouse Alexa Fluor™ Plus 647 (Thermo Fisher Scientific, #A32728TR).

Validation

Custom anti-METTL9 antibody was validated by ELISA; Anti-tag antibody specificity was always confirmed by the lack of signal by ICC and WB in non-tagged cell lines as well as their widespread use in the literature. Other primary antibodies used for immunocytochemistry were chosen for their use in the specialised literature and confirmed by their expected, well defined subcellular localization pattern.

Eukaryotic cell lines

Policy information about cell lines and Sex and Gender in Research

Cell line source(s)	All the mouse Embryonic Stem Cell lines employed in the study were generated starting from the established ES-E14TG2a (derived from a male 129/Ola mouse blastocyst) obtained by ATCC (American Type Culture Collection).
Authentication	The parental cell line was authenticated by morphology by microscope observation, as indicated by ATCC, and by testing its differentiation potential
Mycoplasma contamination	Cells were periodically tested for mycoplasma contamination by PCR
Commonly misidentified lines (See ICLAC register)	None of the lines is present in the ICLAC list

Animals and other research organisms

Policy information about studies involving animals; ARRIVE guidelines recommended for reporting animal research, and Sex and Gender in Research

Laboratory animals	Wild type outbred X. laevis frogs were purchased from Nasco, Fort Atkinson, Wisconsin (USA) and adult females at reproductive stage were used to collect eggs for in vitro fertilization
Wild animals	None

Reporting on sex	Given the very early developmental stage of X. laevis embryos, no sex information is available
Field-collected samples	None
Ethics oversight	Animal use followed ethics recommendations of the European Community (2010/63/UE) and protocol approved by Italian Ministry of Public Health (Auth. #501/2021-PR).

Note that full information on the approval of the study protocol must also be provided in the manuscript.

Plants

Seed stocks	N/A
Novel plant genotypes	N/A
Authentication	N/A
